# Elevated GCN2 levels in cancer cells confer protection from mitotic stress and faster cell movement

**DOI:** 10.1007/s13402-026-01214-5

**Published:** 2026-05-11

**Authors:** Vilte Stonyte, Christina Saeten Fjeldbo, Mélanie Langiu, Michelle Joy-Immediato, Priyanka Swaminathan, Prabin Sharma Humagain, Laura Marian Valencia-Pesqueira, Lilian Lindbergsengen, Coen Campsteijn, Heidi Lyng, Beata Grallert

**Affiliations:** 1 Department of Radiation Biology, Institute for Cancer Research, Oslo University Hospital, Oslo, Norway; 2 Institute of Basic Medical Sciences, Faculty of Medicine, University of Oslo, Oslo, Norway

**Keywords:** GCN2, Cell migration, Mitotic stress, PP1, Cytoskeletal dynamics

## Abstract

**Purpose:**

High expression of the stress-response kinase GCN2 has been linked to poor survival in several cancers including cervical cancer, but its underlying biological roles remain incompletely understood. This study aimed to identify genes whose expression correlates with GCN2 in locally advanced cervical cancer and to use these associations to elucidate cellular functions that could contribute to aggressive disease behavior.

**Methods:**

Correlation analyses were performed between GCN2 expression and genome-wide mRNA profiles in a biobank of 291 patients with locally advanced cervical cancer. Gene ontology and pathway analyses were used to identify enriched biological processes, and functional assays were conducted to validate GCN2 involvement in selected pathways.

**Results:**

GCN2 expression correlated not only with genes involved in cellular stress responses but also with those regulating mitosis and cell migration. Functional analyses confirmed that GCN2 activity promotes both proliferative and migratory capacities, revealing important cancer-relevant roles beyond its canonical function in translational control.

**Conclusion:**

Our findings demonstrate that elevated GCN2 levels support cellular functions that can contribute to tumor aggressiveness. These results suggest that GCN2 plays a direct role in malignant progression and may represent a potential biomarker or therapeutic target in locally advanced cervical cancer.

**Supplementary Information:**

The online version contains supplementary material available at 10.1007/s13402-026-01214-5.

## Introduction

All cells must be able to adapt to changes in their environment to survive in a dynamic environment. Stresses may arise from intrinsic sources (such as mistakes in protein folding, reactive oxygen species) or extrinsic sources (such as shortage of amino acids, hypoxia, damaging agents). One of the most important signaling pathways activated by these stresses is the integrated stress response (ISR), which culminates in reprogramming of translation to restore homoeostasis [1–4]. The ISR consists of several kinases which are each activated by their specific stress types, then phosphorylate the translation-initiation factor eIF2α. This leads to a reprogramming of translation, involving stimulation of translation of the mRNA encoding the transcription factor 4 (ATF4). ATF4, in turn, activates expression of genes involved in cell survival and recovery [5]. Dephosphorylation of eIF2α is thought to signal termination of the ISR and return to normal protein synthesis [6, 7].

Cancer cells are often surrounded by an unfavourable microenvironment, experiencing several kinds of stress, which they have to adapt to in order to successfully proliferate and metastasize. These stresses include endogenous stresses due to oncogene activation and increased translation, as well as exogenous stresses such as a shortage of amino acids and oxygen in a growing tumour, both challenging the ISR [8–11]. The chronic ISR activation not only enables cancer cells to thrive in a hostile environment, but it can also be responsible for treatment resistance [12–14]. Given the importance of the ISR for cancer cells, small-molecule inhibitors to interfere with the ISR are actively developed for clinical application [10, 15, 16].

GCN2 is one of the ISR kinases, which has been known for over 30 years for its importance in the response to amino-acid starvation and other stresses, such as UV irradiation, proteotoxic stress and hypoxia [17, 18]. These stresses are highly relevant in the context of cancer. A number of studies revealed the importance of GCN2 in supporting tumor growth [11, 19–22], which is attributed to its role in stress responses. Recent reports also showed the involvement of GCN2 in shaping the immune response in the tumor microenvironment [23–25], raising interest in GCN2 as a therapeutic target. Furthermore, several recent studies reported activation of GCN2 by an off-target mechanism by other kinases, including agents already in clinical use or trials [26–28], again suggesting that interfering with GCN2 activity could improve the efficacy of other treatments.

However, there are some clues in the literature hinting that the canonical function might not be the only relevant one for cancer. First, a large-scale CRISPR-based study has shown that close to 13% of cancer cell lines (of ca. 2000) are dependent on GCN2 for survival, in contrast to < 1% for each of the other three eIF2-α kinases [29, 30], suggesting that this sensitivity is not associated with the eIF2α kinase activity. Second, the four eIF2α kinases can compensate for each other at least in some model systems [11, 31], which is difficult to reconcile with the concept that the eIF2α kinase activity is the only reason for the importance of GCN2 in cancer. Third, GCN2 has been implicated in controlling ribosome biogenesis independently of its role in the ISR [32]. Fourth, a recent paper reported an ISR-independent role for GCN2 in controlling global translation, and thereby contributing to the maintenance of cellular homeostasis [33]. Fifth, GCN2 has been identified as essential for a cell-cycle checkpoint both in fission yeast and in budding yeast [34–36], pointing to functions in cell-cycle regulation. Furthermore, we recently reported that GCN2 is required for progression through mitosis in human cancer-derived cell lines, through regulation of PP1 activity, and independently of its role in the ISR [37].

Cervical cancer remains a disease with limited therapeutic options at locally advanced stages, highlighting the need to better characterize the molecular pathways driving its progression. Importantly, cervical cancer is one of the tumor types in which high GCN2 expression is associated with poor prognosis. To gain a better understanding of the importance of GCN2 in this disease, we turned to a biobank of cervical-cancer patient samples and explored correlations of GCN2 mRNA levels with that of other genes. Here we show that GCN2 mRNA levels correlate with expression levels of a number of genes, including, but not limited to, genes associated with stress responses. We also identified correlations with genes involved in mitosis, migration and cell motility, and immune responses. Deregulation of selected correlating genes involved in these pathways was also found in cell lines engineered to overexpress GCN2 or depleted for GCN2. Furthermore, we have performed functional studies to investigate the importance of GCN2 overexpression for mitosis and cell migration, functions predicted based on the analysis of patient data. We have shown that not only is GCN2 involved in both processes, but elevated levels of GCN2 confer advantages to cancer cells in the context of these functions.

## Methods

### Gene expression data from clinical samples of cervical cancer

Gene expression data of 291 locally advanced cervical cancer patients treated with curative chemoradiotherapy were used. Patient characteristics, treatment, tumor biopsies and data acquisition have been described previously [38, 39]. Whole genome gene expression data were generated by Illumina Bead Arrays, using total RNA extracted from pretreatment tumor biopsies. Log2-transformed data were used in the analyses. The patients were divided into two cohorts based on the Illumina Bead array version they were assayed by, i.e. WG-6 v3 (cohort 1, *n* = 156) or HT-12 v4 (cohort 2, *n* = 135). An estimate of the background level was calculated based on probes measuring Y chromosome genes [40], and probes where at least 95% of the 291 samples had a signal below this background estimate were excluded. Further, analyses were performed using probes present on both array versions, and with an Entrez gene id annotation, yielding 19,811 probes measuring 14,638 unique genes.

### Identification of genes correlating with GCN2/GCN1

Spearman’s rank correlation analyses of GCN2 or GCN1 gene expression level versus the expression level of all other probes were performed for cohort 1 and 2 separately. The final list of correlating genes was generated as follows: Probes with significant (FDR < 0.05) positive correlation in both cohorts, or negative correlation in both cohorts were included. Genes measured by more than one probe were kept only if all significantly correlating probes correlated with the same direction (i.e. positively or negatively correlating genes). The probe with largest average correlation coefficient (absolute value) for cohort 1 and 2 per gene was kept for the final list of correlating genes. This average correlation coefficient was also used for ranking the correlating genes for each direction.

### Gene set enrichment analysis (GSEA)

Enrichment of the correlating genes in specific biological processes was searched for in the Molecular Signatures Database [41] (MSigDB, v.7.4) Hallmark [42] and gene ontology gene set collections https://tnmplot.com/analysis/. For the list of GCN2-correlating genes (i.e., the 1000 most correlating genes per direction based on the average correlation coefficient in cohort 1 and 2), the genes overlapping with the gene sets were identified, and statistical significance of the overlap was estimated using hypergeometric test.

All analyses of data from clinical samples were performed in R version 4.3.2 [43]. Correction for multiple testing in the spearman correlation analyses and GSEA, was performed according to the Benjamini and Hochberg procedure (FDR).

### Cell culture

The human cervical-cancer cell lines HeLa, Caski, SiHa and LentiX cells were cultivated in DMEM (Dulbecco’s Modified Eagle’s Medium) (Invitrogen) supplemented with 10% fetal bovine serum (FBS) (Gibco) and 1% Penicillin/Streptomycin (P/S) (Gibco). The non-transformed epithelial cell line hTert-RPE1 was cultivated in DMEM/F12 (Invitrogen) supplemented with 10% FBS, 1% P/S and 0.01 mg/ml Hygromycin B (Sigma). All our cell lines are from ATCC. All cell lines were tested negative for mycoplasma contamination and grown at 37 °C in a humidified environment with 20% O_2_ and 5% CO_2_.

Inhibitors were GCN2-IN-1 (GCN2i) (MedChemExpress #HY-100877), Mps1i (MedChemExpress #HY-14710), S-Trityl-L-cysteine (STLC) (Sigma #164739), ISRIB (MedChemExpress #HY-12495).

### Immunoblots

Samples for immunoblots of mammalian proteins were prepared by one wash with cold 1× PBS and kept at -80˚C until 2× Laemmli buffer was added to make whole cell lysates. Alternatively, cells were lysed with lysis buffer (100 mM NaCl, 50 mM Tris (pH 7.5), 2 mM MgCl_2_, 0.5% Triton X-100, 100 nM Calyculin A (LC laboratories), protease inhibitor cocktail (Roche) containing 100 U/ml benzonase (Merck), lysates were cleared by centrifugation at 13 000 rpm for 10 min and mixed with 4 x Laemmli buffer. ECL kits SuperSignal™ Western Blot Substrate Pico PLUS, Femto or Atto were used for detection. Images were captured with the Biorad Chemidoc system, and quantified using ImageLab software. Rabbit Gcn2T899-P 1:500, (R&D Systems, #AF7605); rabbit Gcn2, 1:1000 (Cell Signaling Technology, #3302); rabbit eIF2α-P, 1:1000 (Invitrogen, #44-728G); rabbit GADD34, 1:1000 (Proteintech, #10449-1-AP); rabbit ATF4, 1:500 (Cell Signaling Technology, #11815), rabbit eIF2α, 1:2000 (SCBT, #sc-11386); rabbit GAPDH 1:1000 (Cell Signaling Technology, #5174); mouse γ-tubulin 1:25000 (Sigma, #T6557). Non-cropped immunoblots are provided as a supplementary file.

### Live-cell imaging

Cells were seeded into chambered coverslips (Ibidi, #80806) for analysis of mitotic progression, or into culture inserts (Ibidi, #80366) for wound healing assays. Cells were imaged in Fluorobrite DMEM medium supplemented with 10% fetal bovine serum, 1% penicillin-streptomycin, and Glutamax (ThermoFisher). SPY-DNA-650 (10 nM; SpiroChrome) was used to visualize the nuclei, and SPY555-tubulin (1:5000, Spirochrome) was used to visualize tubulin.

Cells were imaged using a Nikon ECLIPSE Ti2-E inverted microscope (Nikon Corp, Tokyo, Japan) equipped with two Prime BSI sCMOS cameras (Teledyne Photometrics, Tucson, AZ, US), with a CFI Plan Apo l 40 × (0.95 numerical aperture) or a Plan Apo l 10 × (0.45 numerical aperture) objective. Alternatively, a DeltaVision Elite widefield microscope (Applied Precision), equipped with a live cell Elite TruLight Illumination System and a CoolSNAP HQ2-ICX285 camera, using an Olympus UPlan SApo 20 × (0.75 numerical aperture) objective.

Time-lapse images (10 z-Sect. 0.8 μm apart) were acquired every 5 min, or every 20–30 min for wound-healing and chemotaxis assays. Anaphase was scored based on chromosome separation. The microscope stage was kept at 37 °C by a temperature-controlled incubation chamber during live observation.

Images obtained with the Nikon ECLIPSE Ti2-E were Z-projected and analyzed with NIS-Elements AR Analysis software. For DeltaVision imaging, time-lapse images were Z-projected using the softWoRx software (Applied Precision, GE Healthcare). All images were processed using ImageJ for presentation.

### Immunofluorescence

Cells were grown on Precision cover glass and fixed with 4% formaldehyde for 10 min, permeabilized with ice-cold methanol. Primary and secondary antibodies were diluted in PBS containing 5% bovine serum albumin (BSA) and incubated for 1–2 h. After antibody staining, samples were mounted on microscope slides with ProlongGold-DAPI (Invitrogen). Antibodies: rabbit pericentrin 1:400 (Abcam, #ab4448); sheep α/β-tubulin 1:500 (Cytoskeleton Inc ATN02) rabbit Phospho-Myosin Light Chain 2 (Thr18/Ser19) 1:300 (Cell Signaling #3674).

### Transfections

Cells at 50% confluency were transfected with 5 nM siRNA using Lipofectamine RNAiMAX transfection reagent (Life Technologies) following the manufacturer’s instructions. GCN2 targeting siRNA sequence was gcaauucuguggugcauaa.

### Cloning and lentiviral transductions

GCN2 constructs and mutants were as described [37]. Stable cell lines were generated by lentiviral transductions using procedures and plasmids that have been previously described [44]. For the experiments in Fig. 4E and F the destination vector pCW57.1 (Addgene, #41393) was used, which allows doxycycline-regulatable expression of the transgene. Detailed cloning procedures are available on request.

### Migration assays

Wound healing assays were performed either by seeding cells into culture inserts (Ibidi, #80366) and observing them by live-cell microscopy after removing the insert, and images were analyzed using the NIS-Elements AR Analysis software. Alternatively, cells were grown to confluence in 96-well plates, wounded using the Sartorius Wound maker, observed them in an Incucyte, and images were analyzed with the Incucyte Scratch Wound Analysis Software.

For transwell migration assays, cells were pre-incubated in FBS-free medium for 4 h, then seeded into cell culture inserts (Nunc, 8 μm pore size), still in FBS-free medium and exposed to an FBS gradient. After 16 h incubation, not-migrated cells in the chamber were removed, the migrated cells on the underside of the membrane were fixed, stained with DAPI and counted. Cell counts were normalized to the seeding controls, which were determined either by crystal violet staining and counting, or by the CCK cell proliferation assay (Abcam, #ab228554).

Chemotaxis assays were performed using µ-Slide Chemotaxis slides (Ibidi # 80326), live-cell imaging images were analyzed and cells were tracked using the NIS-Elements AR Analysis software, and tracks were analyzed using the Chemotaxis and Migration Tool (Ibidi).

### Attachment assays

20 000 cells were seeded in 8-well Ibidi chambers, incubated for 1 h (RPE) or 2 h (HeLa). Non-attached cells were removed by gentle washing with PBS prior to fixation with 4% formaldehyde. Cells were stained with phalloidin and DAPI and images were analyzed using NIS Elements 6.

### Photoconversion

Photoconversion of actin filaments was performed using a Nikon AXR point-scanning confocal microscope with a NA1.42/60X oil objective and Nikon NIS-Elements software (Nikon Instruments Inc., Tokyo, Japan). A 405 nm laser was used at 0.5%, focused on a small ROI in the middle of an actin stress fiber (diameter ~ 1 μm), with a dwell time of 1 µs. Images were acquired before and after photoconversion, every 10 s for 4 min.

For each cell, mean fluorescence intensities were measured in both the green (unconverted) and red (photoconverted) channels at each timepoint, within the ROI used for photoconversion. Background fluorescence was determined from a cell-free region in each frame, using a second ROI of the same size, and subtracted from both channels. Photoconversion was quantified as the fraction of red fluorescence relative to total fluorescence, calculated as *R/(R + G*) where *R* and *G* represent background-corrected intensities in the red and green channels, respectively. Curve fitting was performed on a per-cell basis using nonlinear regression in GraphPad Prism. The resulting rate constants (*k*) were used for statistical comparison between conditions.

### Mass spectrometry

For BioID, cells expressing GCN2-UltraID or Ultra ID [45] were labelled for 10 min with 50 µM biotin, then washed with PBS with 10 mM sodium azide and 10 mM EDTA. Cells were harvested and lysed in lysis buffer (20 mM Tris pH-7.5, 0.1% Triton X-100, 50 mM NaCl, cOmplete protease inhibitors (Roche), phosSTOP (Roche)). Streptavidin beads (ThermoFisher, #88816*)* were incubated overnight with the lysate to immunoprecipitate biotinylated proteins. Beads were washed three times with wash buffer I (20 mM Tris pH-7.5, 0.05% Tween-20, 50 mM NaCl, 0.5% SDS, 0.5% Na-deoxycholate, cOmplete protease inhibitors (Roche), phosSTOP (Roche)), then once with wash buffer II (2 M urea + 10 mM Tris + 1 mM EDTA + 0.1% Triton X-100), and finally twice with PBS. Samples were analyzed by a nanoElute UHPLC coupled to a tims-TOF Pro 2 mass spectrometer (Bruker Daltonics, Bremen, Germany) via a CaptiveSpray ion source. The mass spectrometer was operated in data-independent Parallel Accumulation-Serial Fragmentation (PASEF) mode at the Proteomics Core Facility at Oslo University Hospital.

For PP1γ IP-s, cells carrying GFP-tagged PP1γ were lysed in lysis buffer (20 mM Tris pH-7.5, 0.1% Triton X-100, 50 mM NaCl, cOmplete protease inhibitors (Roche), phosSTOP (Roche), 100 nM Calyculin A) and GFP-trap (ChromoTek #GTMA) beads were used to immunoprecipitated PP1γ-GFP. Samples were analyzed by a nanoLC system coupled to a Q Exactive mass spectrometer (Thermo Fisher Scientific, Bremen, Germany) equipped with a nano-electrospray ion source at the Proteomics Facility at the University of Oslo.

The actin-related gene lists (Table S5) were generated from MSigDB Gene Ontology collections (GO biological process): ADHERENS_JUNCTION_ASSEMBLY, CELL_MOTILITY, ACTIN_FILAMENT_BASED_MOVEMENT, ACTIN_FILAMENT_BUNDLE_FORMATION, ADHERENS_JUNCTION_ORGANIZATION, and ADHERENS_JUNCTION_MAINTENANCE [41, 42], and were further manually curated based on literature on actin nucleation, elongation and turnover [46–54].

## Results

### GCN2 expression levels correlate with that of genes involved in cancer-relevant processes

**Fig. 1:**
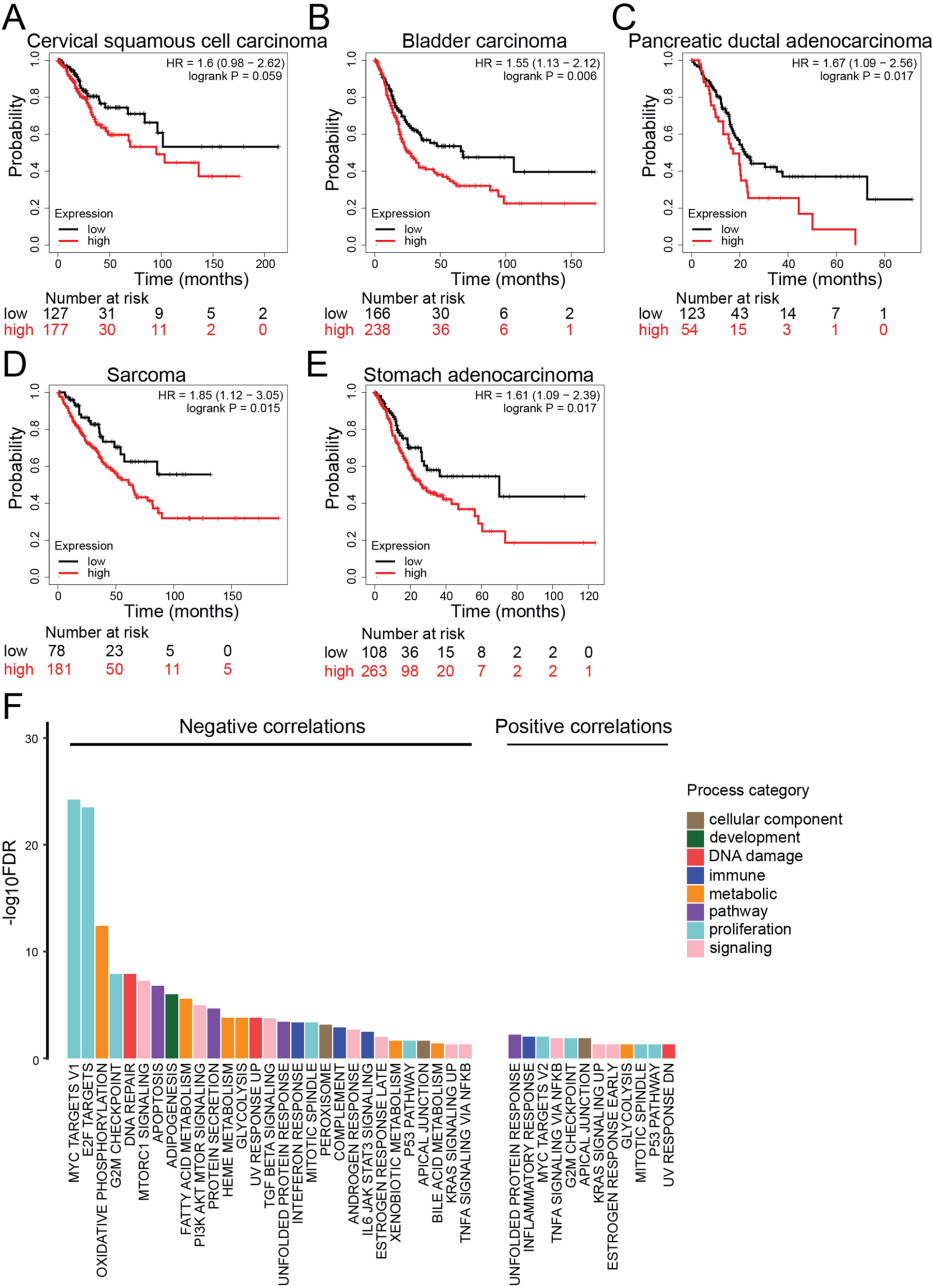
Elevated GCN2 expression levels correlate with poor prognosis across multiple cancers. **A-E** Kaplan-Meier plots were generated in https://www.kmplot.com/analysis/ [55]. Probability of overall survival is shown for various patient cohorts using gene expression data in the GEO or TCGA data bases. The patients were split into two groups by the cutoff expression value that provided the highest significance. **F** GSEA of GCN2-correlating genes. Enriched gene sets (FDR < 0.05) from the MSigDB Hallmark gene set for negatively (left) and positively (right) correlating genes. Selected gene sets indicating possible new functions of GCN2 pursued in this study are shown in bold. The results of the GSEA of the top-ranked 1000 correlating genes in each direction are shown in Table S3

Consistent with the idea that GCN2 contributes to cancer development, elevated GCN2 mRNA levels correlate with poor prognosis in several cancers [55, 56], including cervical cancer (Fig. 1A-E). To explore biological processes associated with GCN2, we analyzed whole-genome expression data from two independent cohorts of patients with cervical cancer (cohort 1, *n* = 156; cohort 2, *n* = 135). Spearman’s rank correlation analysis between GCN2 mRNA levels and all other genes identified 2629 and 1179 genes showing significant (FDR < 0.05) negative and positive correlations with GCN2 in both cohorts, respectively (Table S1).

To validate our approach, we first examined correlations expected from the canonical function of GCN2. GCN1, a ribosome-associated factor required for GCN2 activation in the integrated stress response (ISR), transmits signals from stalled ribosomes upon starvation or other stresses [17, 57–61]. Given their close functional relationship, an overlap between genes correlating with GCN2 and GCN1 mRNA levels was anticipated. Indeed, GCN2 levels correlated significantly with GCN1 levels in both cohorts (Fig. S1A), and 2231 (59%) of the 3798 genes correlating with GCN2 also correlated with GCN1 (Fig S1B). In addition, 40 of 109 previously identified ATF4 target genes [11, 62] were found among the genes correlating with GCN2 and/or GCN1 in the patient data set (Table S2). The identification of these expected correlations validates our approach.

We next investigated which biological processes were enriched among GCN2-correlating genes by subjecting the 1000 most positively and 1000 most negatively correlating genes to gene set enrichment analysis (GSEA) (Fig. 1F, Table S3). This analysis revealed biological processes previously associated with GCN2 and the ISR, including the Myc pathway [11, 63], TGF-β signaling [30], oxidative phosphorylation [64, 65], inflammatory signaling [23, 66–68] and DNA repair [69]. Intriguingly, GSEA also highlighted hallmarks not typically associated with GCN2, such as “mitotic spindle,” “G2/M checkpoint,” and “apical junctions”, suggesting potential novel functions for GCN2.

To further delineate these associations, we analysed Gene Ontology (GO) terms associated with the GCN2-correlating genes overlapping with these hallmarks. The enriched GO terms included positive regulation of cell migration and cell motility, regulation of actin filament polymerization, and mitotic spindle assembly (Fig S2, Table S4). Moreover, comparison of the GCN2-correlating genes with the MSigDB C5 GO BP database identified 94 positively correlating genes annotated with the GO term “locomotion*”*, 87 of which were also associated with “cell migration*”*. Together, these analyses associate GCN2 expression with genes involved in cancer-relevant processes such as mitotic control, cell motility, and cytoskeletal organization.

To further probe the functional significance of these correlations, we selected representative genes from each pathway of interest and examined whether their mRNA levels changed upon GCN2 modulation in isogenic cell lines, where GCN2 was either depleted by siRNA (siGCN2) or overexpressed (GCN2^high^) in HeLa (of cervical-cancer origin) and hTert-RPE1 cells (non-transformed epithelial cell line). Immunoblotting confirmed efficient knockdown and overexpression of GCN2, with signal intensities compared against a serial dilution of control lysates to allow visual estimation of depletion efficiency (Fig S3A, B). Expression analysis revealed modest alterations of the selected genes upon GCN2 depletion or overexpression (Fig S3C, D). The direction and magnitude of these changes varied between the two cell lines.

To evaluate these associations from the patient dataset on a broader scale, we turned to a large pan-cancer transcriptomics database [29] to query correlations between GCN2 expression and expression of the same set of genes. Most correlations were recapitulated across multiple cancer types (Table 1), and in cases where a specific gene did not correlate, a functionally related component of the same complex or pathway did so. Although these mRNA-level trends and correlations cannot be interpreted as direct regulatory relationships, they strongly support a broader involvement of GCN2 in processes such as mitotic control and cell migration. In this scenario, altered GCN2 levels may affect these processes, thereby influencing the expression of genes associated with the given pathway. Guided by these observations, we next performed functional analyses to directly assess the impact of GCN2 on these processes.

**Table 1:** Correlations of selected genes with GCN2 in DepMap

Correlations of the selected genes in DepMap			Significantly correlating functional relatives in DepMap of genes that correlate with GCN2 in patient data but not in DepMap		
Gene	Pearson	p-value	Gene	Pearson	p-value
FLNA	0.447	7.07E-70			
GSN	0.296	6.58E-30			
NEK2	0.042	1.16E-01	NEK7	0.281	7.80E-27
CTTN	0.275	9.38E-26			
MAD2L1	0.032	2.26E-01	MAD3L	0.154	5.97E-09
SMC3	0.061	1.81E-02	SMC2 SMC5	0.131 0.125	3.99E-07 1.36E-06
NET1	-0.106	6.85E-05			
TUBGCP6	-0.091	6.40E-04			
*n* = 1406					

Genes identified as correlating in the biobank are shown in bold. When genes correlated in the patient data but not in DepMap, functional relatives were also queried and significant correlations are shown on the right

### High GCN2 levels protect cells from some forms of mitotic stress

**Fig. 2:**
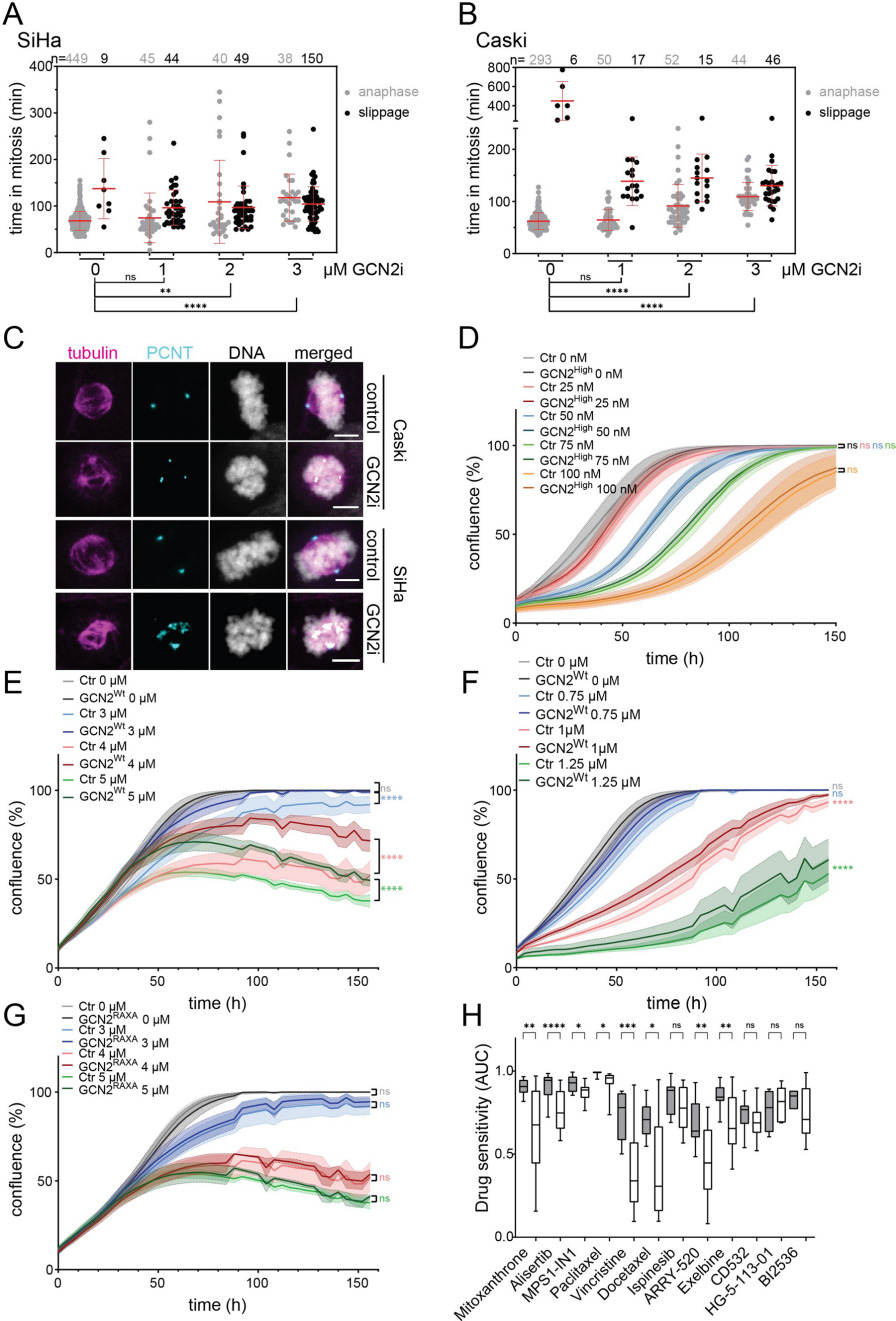
High GCN2 levels protect cells from some forms of mitotic stress. **A**,** B** Progression through mitosis in the presence of GCN2i at the indicated concentrations was observed by live-cell imaging in (**A**) SiHa and (**B**) Caski cells. The time in mitosis is shown for each cell. Prophase was judged by the first frame showing chromatin condensation, anaphase was scored based on chromosome separation and mitotic slippage was scored based on decondensation of the DNA in the absence of chromosome separation. Brown Forsythe and Welch Anova test was performed on the total time in mitosis. SiHa 0 µM compared to 1 µM *p* = 0.1236; 2 µM *p = 0.0014; 3 µM ****p < 0.0001. Caski 0 µM compared to 1 µM *p* = 0.0551; 2µM and 3 µM **** *p* < 0.0001. **C** Caski and SiHa cells were incubated in the absence (control) or presence (GCN2i) of GCN2i for 8 h and fixed for immunofluorescence. Representative images of mitotic cells stained for tubulin (magenta), pericentrin (PCNT, cyan) and DNA (grey) are shown. Scale bars represent 5 μm. **D** High levels of GCN2 do not protect starved cells. HeLa cells transduced to overexpress GCN2 (GCN2^high^) and the parental cell line (Ctr) were grown in the presence of HFG at the indicated concentrations and observed in Incucyte. Data shown are from three independent experiments. Mean ± SEM are shown, non-linear regression, *p* = 0.9867 for 0 nM, *p* = 0.1607 for 25 nM, *p* = 0.9012 for 50 nM, *p* = 0.1994 for 75 nM, *p* = 0.3283 for 100 nM. **E** High levels of GCN2 protect cells exposed to MPS1i. HeLa cells transduced to overexpress GCN2 (GCN2^WT^) and the parental cell line (Ctr) were grown in the presence of MPS1i at the indicated concentrations and observed in Incucyte. Data shown are from four independent experiments. Mean ± SEM are shown, non-linear regression, *p* = 0.3393 for 0 µM, ****p < 0.0001 for 3 µM, 4 µM, and 5 µM. **F** High levels of GCN2 protects cells exposed to Eg5i. HeLa cells transduced to overexpress GCN2 (GCN2^WT^) and the parental cell line (Ctr) were grown in the presence of Eg5i at the indicated concentrations and observed in Incucyte. Non-linear regression, *p* = 0.3393 for 0 µM, *p* = 0.0712 for 0.75 µM, ****p < 0.0001 for 1 µM and 1.25 µM. **G** Protection in cells exposed to MPS1i is lost in cells expressing GCN2^RAXA^. HeLa cells transduced to express siRNA-resistant GCN2^RAXA^ were transfected with GCN2-targeting siRNA, grown in the presence of MPS1i at the indicated concentrations, and observed in Incucyte. Data shown are from four independent experiments. Mean and SEM are shown, non-linear regression, p = 0.5069 for 0 µM, p = 0.1346 for 3 µM, p = 0.4104 for 4 µM, p = 0.4254 for 5 µM. Note that the data shown for control cells are the same as those shown in Fig. 2E. **H** 10% of the cell lines with the highest and lowest GCN2 levels were selected in the DepMap database (see Fig S3G), and their sensitivity (AUC) to drugs in the Sanger GDSC1 and GDSC2 datasets is shown. Grey boxes represent the cell lines with GCN2 levels in the top 10%- (GCN2^High^), white boxes represent cell lines with GCN2 levels in the bottom 10% (GCN2^Low^). Whiskers represent min to max values. Welch’s two-tailed t-test, p values are 0.002 for mitoxanthrone, ****p < 0.0001 for alisertib, 0.0174 for MPS1-IN1, 0.0268 for paclitaxel, 0.0007 for vincristine, 0.0119 for docetaxel, 0.0962 for ispinesib, 0.0020 for ARRY-250, 0.0064 for exelbine, 0.2231 for CD532, 0.4972 for HG-5-113-01, 0.1211 for BI2536

G2/M checkpoint and mitotic spindle were hallmarks associated with several genes correlating with GCN2 mRNA levels, pointing to a function in mitosis. This finding further confirms and supports our recent report where we identified PP1α and PP1γ as direct GCN2 substrates, and showed that GCN2 restrains PP1 activity to ensure timely phosphorylation of key mitotic regulators such as AURKA, CENPE, and TACC3 [37]. While these findings highlight the relevance of GCN2–PP1 signalling for chromosome alignment and spindle function, the full complement of PP1 substrates regulated by GCN2 remains to be defined. Remarkably, this function was not essential for normal mitotic progression in the non-transformed hTert-RPE1 cell line, but was essential in several cancer cell lines, including HeLa of cervical cancer origin [37]. We further investigated the impact of GCN2 inhibition on mitosis in other cervical-cancer-derived cell lines. In all experiments involving a GCN2 inhibitor we used GCN2-IN-1, referred to as GCN2i throughout the paper. SiHa and Caski cells also had a severe mitotic phenotype upon inhibition of GCN2 and could not align their chromosomes in a metaphase plate (Fig. 2A, B). Most HeLa cells eventually performed anaphase in spite of chromosome alignment defects and mitotic slippage was less penetrant [37]. Here we find that in SiHa and Caski cells fewer cells were able to perform anaphase and instead underwent mitotic slippage (as judged from condensing and then decondensing their chromosomes) (Fig. 2A, B). Closer inspection revealed that > 80% of the cells undergoing “slippage” displayed spindle collapse, including multipolar spindles (Fig. 2C, Movies 1–4), which was likely the reason for their failure to separate their chromosomes into two daughter nuclei. Notably, multipolar spindles were also observed in HeLa cells [37], even if this particular mitotic phenotype was less penetrant. While the exact outcome depends on the interplay of several regulatory mechanisms, which are deregulated in different ways and extents in different cancer cells, a common theme is that inhibiting GCN2 activity in cervical-cancer cells interferes with mitotic progression (Fig. 2A-C and [37]) and leads to reduced cell viability (Fig S3E and [37]).

The involvement of GCN2 in mitosis raises the question whether it is important in the context of the correlation of high GCN2 levels with poor survival. To test the impact of high GCN2 levels on cell growth under stress, we considered both the canonical role under amino-acid starvation and the mitotic role. To test whether high levels of GCN2 confer growth advantages under starvation conditions, we grew HeLa cells and GCN2^high^ HeLa cells in the presence of halofuginone (HFG), an inhibitor of glutamyl-prolyl tRNA synthetase, and monitored cell growth. Surprisingly, high levels of GCN2 did not confer a growth advantage in face of starvation (Fig. 2D). In light of the novel function in mitosis, a plausible hypothesis is that high levels of GCN2 can protect the cells from some forms of mitotic stress. To test this hypothesis, we exposed HeLa and GCN2^high^ HeLa cells to inhibitors affecting mitotic processes. When cells were exposed to an inhibitor of the SAC kinase MPS1, high GCN2 levels led to a significantly improved survival (Fig. 2E, GCN2^WT^). A similar trend was observed using STLC, an inhibitor of the mitotic kinesin Eg5 / KIF11 (Fig. 2F). Notably, overexpression of a GCN2^RAXA^ mutant that cannot bind PP1 [37] did not confer this growth advantage in the presence of the MPS1 inhibitor (Fig. 2G, Fig S3F), confirming that this effect is due to GCN2’s mitotic function through PP1 regulation. These results support the notion that high levels of GCN2 confer growth advantages under certain forms of mitotic stress in cancer cells. To assess this suggested link between high GCN2 levels and protection from mitotic stress, we analysed data from to the DepMap database. Cell lines were ranked according to GCN2 expression, and drug sensitivity of those in the highest and lowest deciles (top and bottom 10% of 1674 lines; Fig S3G) was compared in the Sanger GDSC1 and 2 datasets. Remarkably, sensitivity to most (4 out of 6 of the mitotic drugs in both datasets was significantly higher in the “GCN2 low” group than in the “GCN2 High” group (Fig. 2H). Next, we queried the CellMiner database (discover.nci.nih.gov/cellminercdb) [70] to further assess drug responses as a function of GCN2 expression levels. A trend of higher GCN2 levels correlating with higher IC50 values for several mitotic drugs was observed also in this dataset (Fig S4). Although the correlations were generally weak, they reached significance for several Aurora kinase inhibitors and MPS1-IN1, but not for most drugs interfering with microtubule function. Together, these analyses support our data suggesting that elevated GCN2 levels may protect cancer cells from certain forms of mitotic stress.

**Fig. 3:**
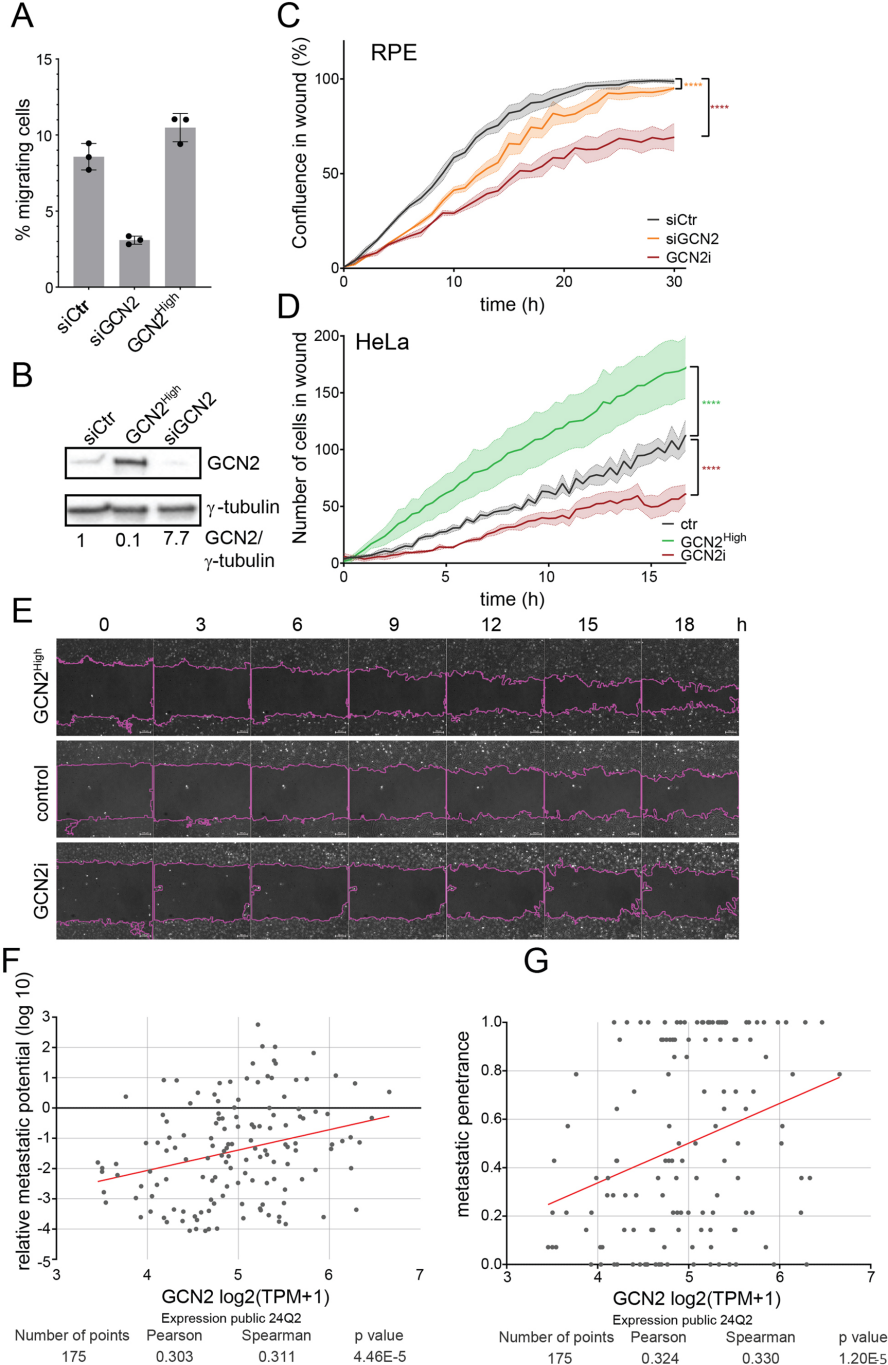
GCN2 promotes cell migration. **A**, **B** hTert-RPE1 cells were transfected with GCN2-targeting siRNA for 48 h (siGCN2) or transduced to stably overexpress GCN2 (GCN2^High^). **A** Cells were seeded without FBS into transwell chambers with 8 μm pore size and exposed to an FBS gradient for 16 h. Migrated cells were counted after DAPI staining and normalized to seeding controls. Three independent experiments, mean and STDEV are shown. One-way Anova, ** *p* = 0.0065, *p = 0.0109 **B** GCN2 levels and knock-down efficiency in the experiment shown in A. γ-tubulin is used as a loading control **C** hTert-RPE1 cells were transfected with GCN2-targeting siRNA (siGCN2) or treated with 2 µM GCN2i. Cells were grown to confluence and wounds were created using a Wound Maker tool (Sartorius) and healing was monitored in Incucyte. Three (siGCN2) or five (GCN2i) independent experiments, mean ± SEM are shown, non-linear regression, ****p < 0.0001. GCN2i at this concentration abolishes the canonical function of GCN2 (shown in Fig S5E). **D** HeLa cells treated with 2 µM GCN2i or transduced to stably overexpress GCN2 (GCN2^high^) were seeded into Ibidi culture inserts and grown to confluence. After removal of the inserts the cells were observed by live-cell imaging. The number of cells migrating into the initial wound area is shown. Three (GCN2i) or four (GCN2^High^) independent experiments, mean and SEM are shown, non-linear regression, ****p < 0.0001. GCN2i at this concentration abolishes the canonical function of GCN2 (shown in Fig S5F). **E** Representative images from an experiment shown in (**D**). **F**,** G** Metastatic potential (**F**) and penetrance (**G**) in metastatic cancers as a function of GCN2 mRNA levels. Data were downloaded from depmap.org and are based on a study by Jin et al. (2020), which reported metastatic-potential profiling of ca. 500 human cancer cell lines derived from 21 types of solid tumour in immunodeficient murine models [72]. Metastatic potential is calculated based on the mean cancer-cell numbers detected in the target organs. Metastatic penetrance refers to the proportion of mice displaying metastases with the given cell line [72]

**Fig. 4:**
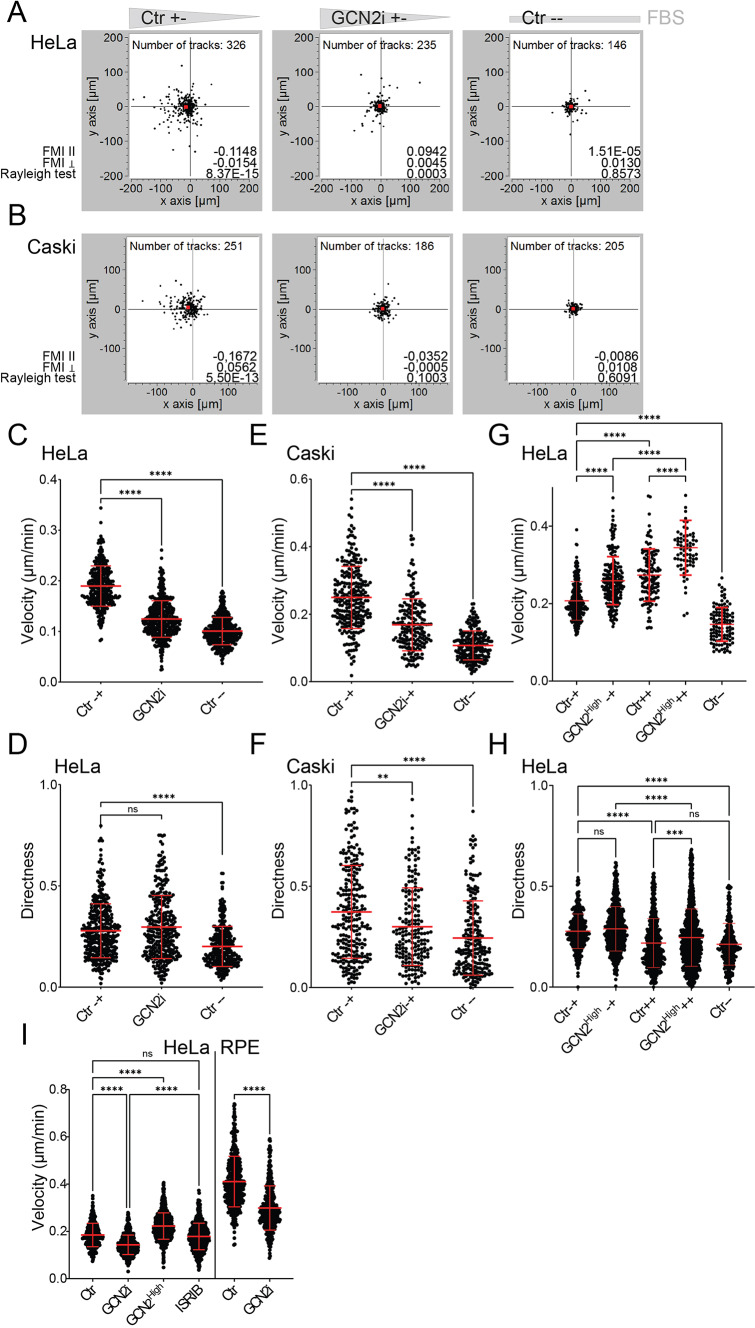
GCN2 regulates cell movement. **A**,** B** HeLa (**A**) or Caski (**B**) cells were seeded into Ibidi µ-Slide Chemotaxis with or without an FBS gradient and observed by live-cell imaging. Shown are endpoints of tracks from representative experiments. Red squares indicate the center of mass displacement. +- denotes the presence of FBS gradient, -- denotes no FBS on either side; the concentration of FBS is also indicated by the grey shaded triangles. Forward Migration Index (FMI) indicate the cells’ displacement parallel (FMI ll) or perpendicular (I) to the gradient. Negative FMI values represent movement toward higher FBS concentrations. The significance of directional bias was evaluated using the Rayleigh test, which assesses deviation from random migration. **C**,** E**,** G** Velocity of the indicated cell lines and treatments was determined in chemotaxis experiments in three (**C**,** G**) or two (**E**) independent experiments. -+ denotes the presence of FBS gradient, -- denotes no FBS on either side. Cumulative results are shown. Welch’s two-tailed t test, ****p < 0.0001. **D**,** F**,** H** Directness of movement of the indicated cell lines and treatments was determined in chemotaxis experiments in three (**D**,** H**) or two (**F**) independent experiments. -+ denotes the presence of FBS gradient, -- denotes no FBS on either side. Cumulative results are shown. Welch’s two-tailed t test, *p* = 0.0963 for GCN2iversus Ctr -+; ****p < 0.0001 for Ctr -+ versus Ctr --. **I** Velocity of the indicated cell lines was determined in standard growth medium, without any gradient, in three (HeLa) or five (hTert-RPE1) independent experiments. Cumulative results are shown. One-way Anova, **** *p* < 0,0001; ns *p* = 0.7052

**Fig. 5:**
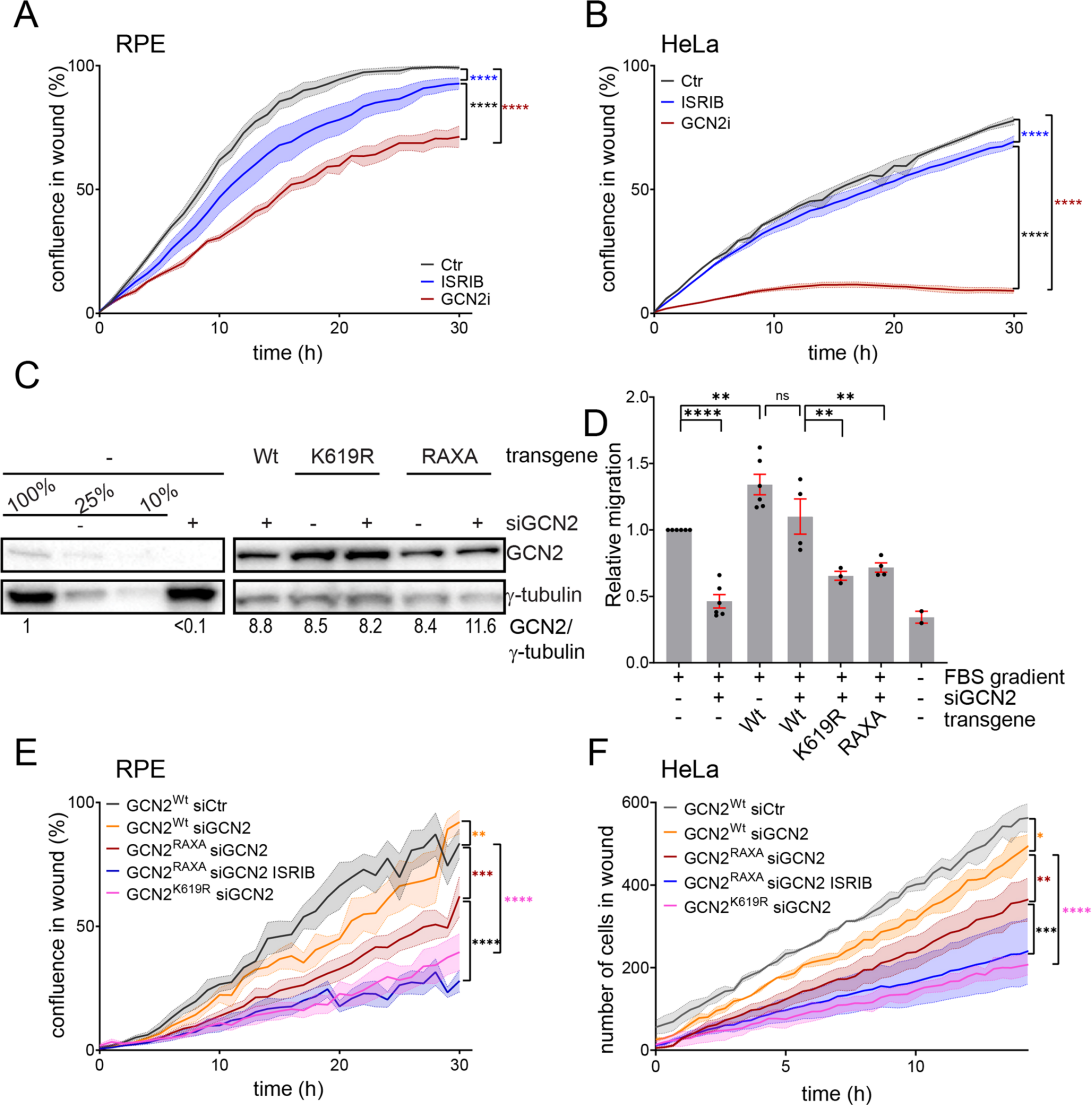
GCN2-dependent migration requires eIF2α phosphorylation and PP1 regulation. **A**,** B** hTert-RPE1 (**A**) and HeLa (**B)** cells were grown to confluence and wounds were created using a Wound Maker tool (Sartorius) and healing was monitored in Incucyte. ISRIB or GCN2i was added to 200 nM or 2 µM, respectively. Four (hTert-RPE) or three (HeLa) independent experiments, mean and SEM are shown, non-linear regression, ****p < 0.0001. **C**,** D** hTert-RPE1 cells transduced with siRNA-resistant GCN2 carrying the indicated mutations were transfected with control or GCN2-targeting siRNA for 48 h. (**C**) Knock-down efficiency and expression of the mutant transgenes in the experiments shown in D was tested by immunoblotting. Different exposures of the same blot are shown for GCN2. γ-tubulin is shown as loading control. **(D)** Cells were seeded without FBS into transwell chambers with 8 µM pore size and exposed to an FBS gradient for 16 h. Migrated cells were counted after DAPI staining and normalized to seeding controls and then to migration in cells transfected with control siRNA. Mean ± SEM are shown, results from at least three independent experiments. One-way Anova, ****p < 0.0001 siCtr versus siGCN2; **p = 0.0022 siCtr versus GCN2^Wt^ siCtr; **p = 0.0051 GCN2^Wt^ siGCN2 versus GCN2^RAXA^ siGCN2; **p = 0.0025 GCN2^Wt^ siGCN2 versus GCN2^K619R^ siGCN2. **E** hTert-RPE1 cells transduced with doxycycline-inducible siRNA-resistant GCN2 carrying the indicated mutations were transfected with control (siCtr) or GCN2-targeting siRNA (siGCN2) and grown to confluence in the presence of doxycycline. Wounds were created using a Wound Maker tool (Sartorius) and healing was monitored in Incucyte. ISRIB was added to 200 nM after wounding. Averages (lines) and SEM (bands) from six independent experiments are shown. Non-linear regression, **p = 0.0028, ***p = 0.0002, **** *p* < 0.0001. **F** HeLa cells transduced with doxycycline-inducible siRNA-resistant GCN2 carrying the indicated mutations were transfected with control (siCtr) or GCN2-targeting siRNA (siGCN2), seeded into Ibidi culture inserts, and grown to confluence in the presence of doxycycline. After removal of the inserts the cells were observed by live-cell imaging. ISRIB was added to 200 nM after removal of the inserts. The number of cells migrating into the initial wound area is shown. Averages (lines) and SEM (bands) from three independent experiments are shown. Non-linear regression, ****p < 0.0001, *p = 0.0113, **p = 0.0072, ***p = 0.0006

### High GCN2 levels promote cell migration

#### GCN2 regulates cell motility through eIF2α and PP1

Our analyses also revealed a potential link between GCN2 and cell migration and motility, prompting us to investigate whether GCN2 influences these processes. We assessed the cells’ ability to move towards a chemoattractant in a transwell migration assay, using FBS as a chemoattractant, employing isogenic cell lines overexpressing (GCN2^high^) or depleted for GCN2 (siGNC2) (Fig. 3A, B). The capacity for directional migration correlated with GCN2 levels: not only did cells have a reduced capacity when depleted for GCN2, but, remarkably, cells overexpressing GCN2 had an increased capacity for directional migration.

To further explore the relevance of GCN2 function for cell migration using an independent assay, we performed wound-healing assays using hTert-RPE1, HeLa, and Caski cells. In the absence of GCN2 function migration into the gaps was severely impaired in all three cell lines (Fig. 3C, D, Fig S5A), consistent with a previous observation using keratinocytes [71]. Notably, GCN2 is not essential for mitosis in RPE cells [37], further supporting the notion that the observed impaired wound healing is not a result of impaired cell division but of impaired cell migration. In HeLa cells, treatment with GCN2i leads to mitotic errors and cell death [37], which could influence the wound-healing ability. However, even if mitotic delays might contribute to the observed wound-healing defect, cell death in the time scale of the wound-healing assay is negligible (Fig S5B). Furthermore, HeLa cells engineered to overexpress GCN2 (GCN2^high^) were able to close the gap faster than control cells (Fig. 3D, E, Movies 5–7), suggesting that high levels of GCN2 in cancer cells lead to higher capacity to migrate. This finding raises the possibility that elevated GCN2 levels observed in patients may enhance the migratory capacity of cancer cells, potentially promoting metastatic dissemination and contributing to the association between high GCN2 expression and poor clinical outcome. To further explore the idea that metastatic potential correlates with elevated GCN2 levels, we queried the Metastasis Map (MetMap) database, which describes organ-specific metastasis patterns of ca. 500 human cancer cell lines in immunodeficient murine models [72]. In this sample set GCN2 expression levels clearly correlate with increased metastatic potential (Fig. 3F) and penetrance (Fig. 3G). Consistently, comparing the metastatic potential and penetrance of cell lines with “low” and “high” GCN2 expression levels (Fig S3G) reveals a modest but significant difference (Fig S5C, D).

Directional cell movement requires perceiving and interpreting spatial signals, as well as rearranging the cytoskeleton to achieve movement. In order to address whether the loss of ability to respond to spatial cues is the reason for the impaired directional movement, we used a chemotaxis assay where cells were seeded in medium without FBS, challenged with an FBS gradient, and observed by live-cell imaging. Directional migration was quantified using the Forward Migration Index (FMI), where higher FMI values reflect more pronounced movement along the gradient direction. The Rayleigh test was used to assess whether the observed movement deviated from random migration. In the absence of GCN2 activity both HeLa and Caski cells had reduced ability for chemotactic movement (Fig. 4A, B), consistent with the results of the wound-healing and transwell assays. Remarkably, the speed of movement was considerably impaired in the absence of GCN2 function in both cell lines, (Fig. 4C, E), while the directness of movement was only modestly affected (Fig. 4D, F). Furthermore, overexpression of GCN2 in HeLa cells, which led to faster wound healing (Fig. 3D, E), correlated with increased cell velocity (Fig. 4G) rather than with increased directness of movement (Fig. 4H). These results suggest that the primary reason for poor wound healing or chemotactic movement in the absence of GCN2 is not an impaired ability to respond to spatial cues, but rather impaired movement, and faster wound healing in cells with elevated GCN2 levels correlates with faster movement. To further explore this conclusion, we also assessed cell velocity under normal growth conditions where the cells were not exposed to any gradient or wound. Consistently with the findings of the chemotaxis assays, cell movement was affected by GCN2 inhibition in both HeLa and RPE cells and cells overexpressing GCN2 had a higher velocity (Fig. 4I). These data strongly suggest that GCN2 affects directional cell migration through affecting cell movement, although a smaller impact on directness of movement cannot be excluded.

Previous studies implicated the canonical function of GCN2 in wound healing [65, 71]. In order to explore whether eIF2α is the only relevant substrate in this function, we made use of ISRIB, a drug that binds elF2B and thereby antagonizes the inhibitory effect of phosphorylated eIF2α [73] and compared its effects on wound healing to that of GCN2 inhibition. ISRIB impaired the wound-healing capacity of the cells, consistent with an involvement of eIF2α phosphorylation and the results of the previous reports. Interestingly, GCN2 inhibition had a more severe effect in RPE, HeLa (Fig. 5A, B) and Caski (Fig S5A) cells than treating the cells with ISRIB, using drug concentrations that abolish the starvation-induced induction of GADD34 (Fig S5E, F). These results suggest that additional substrate(s) are important for this function. We recently identified PP1α and γ as novel GCN2 substrates and showed that a GCN2^RAXA^ mutant, unable to bind PP1 PP1α and γ, is still able to phosphorylate eIF2α [37]. To address the importance of PP1 binding we employed stable cell lines depleted for the endogenous GCN2 by siRNA, and expressing siRNA-resistant wild-type (GCN2^WT^), kinase-dead (GCN2^K619R^) or RAXA-mutant GCN2 (GCN2^RAXA^) (Fig. 5C, S5G, H). Neither mutant could rescue the migration defect seen in the absence of endogenous GCN2 in (Fig. 5D, E, F), and the effect of ISRIB was additive with the effect of the RAXA mutation (Fig. 5E, F). These data suggest that eIF2α, and PP1α and/or PP1γ are both relevant for the function of GCN2 in migration.

### GCN2 influences cytoskeletal dynamics and cell attachment

**Fig. 6:**
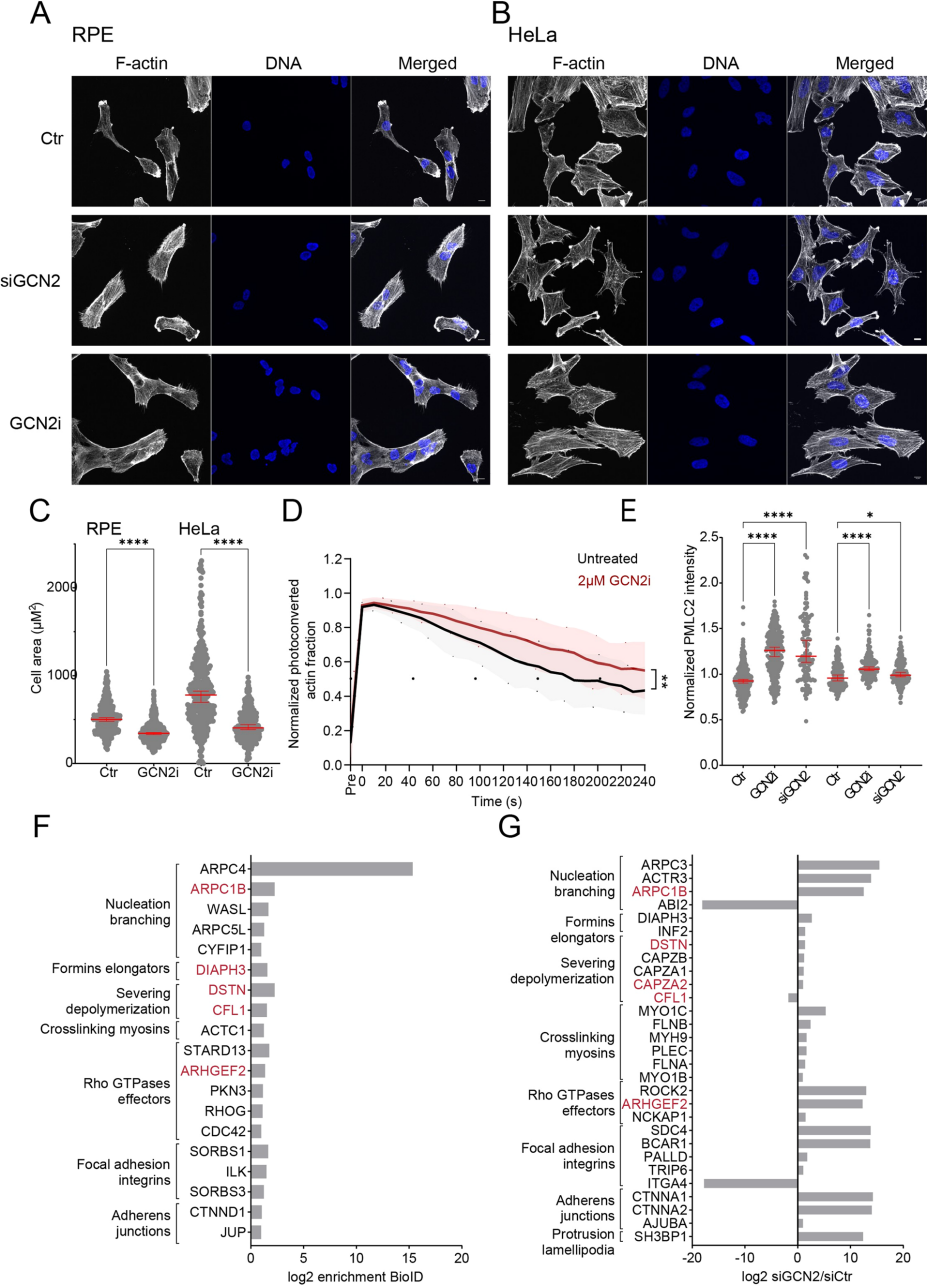
GCN2 has a role in cytoskeletal regulation. **A**, **B** Cells subjected to the indicated treatments were fixed 24 after seeding into Ibidi 8-well chambers with 4% formaldehyde, and stained with phalloidin and DAPI. Representative images are shown. Scale bars represent 10 µM. **C**, Cells adhered 1 h (RPE) or 2 h (HeLa) after seeding were stained with DAPI and phalloidin. Area of the cells as defined by phalloidin staining was measured using NIS elements 6. The violin plots show the distribution of single-cell values; each dot represents one cell. Red lines indicate the median and 95% confidence interval. Brown-Forsythe and Welch Anova test; **** *p* < 0.0001. **D**, Photoconverted actin decay rates were quantified over 4 min in photoconversion experiments using RPE1 mEos2-actin cells under control conditions (black) or following treatment with 2 µM GCN2i (red). *N* = 10 cells per replicate, 2 replicates per condition. Mean and SD are shown, **p = 0.0022; Mann-Whitney U test. **E**, RPE and HeLa cells were subjected to the indicated treatments, fixed with 4% formaldehyde, and stained with an antibody against PMLC2, phalloidin and DAPI. PMLC2 intensity was quantified in cellular masks defined based on actin staining and normalized to the mean nontreated control value. The violin plots show the distribution of single-cell values; each dot represents one cell. Red lines indicate the median and 95% confidence interval. Brown-Forsythe and Welch Anova test; **** *p* < 0.0001; * *p* = 0.0397. **F**, BioID proximity-labeling comparing GCN2–BioID to ligase-only control. Proteins enriched *≥* 2-fold in the GCN2-BioID sample are shown. **G**, PP1 co-immunoprecipitation followed by mass spectrometry in cells treated with siGCN2 or siCtrl. Proteins with *≥* 3-fold change in the siGCN2 relative to siCtr are displayed. Proteins detected in both datasets are highlighted in red

Because efficient cell migration requires coordinated regulation of actin dynamics and adhesion, we next examined whether loss of GCN2 function affects these processes. Phalloidin staining revealed clear differences in F-actin organization between control cells and cells treated with GCN2i or depleted of GCN2 in both cell lines (Fig. 6A, B). Control cells displayed prominent lamellipodia, whereas treated cells exhibited narrower, more discontinuous lamellipodia, accompanied by numerous thin, filopodia-like protrusions and an overall increase in punctate F-actin signal. Cell attachment assays further showed that inhibition of GCN2 altered the ability of cells to adhere to the substrate (Fig. 6C, Fig. S6A).

To directly assess actin dynamics, we monitored actin turnover using photoconvertible actin. Monitoring dynamics of locally photoconverted mEOS-Actin showed that treatment with GCN2i resulted in a modest but significant reduction in actin turnover in stress fibers (Fig. 6D). Consistently, the intensity of phospho-myosin light chain 2 (PMLC2), a readout of myosin II–dependent actomyosin contractility [74], was higher in cells treated with GCN2i or depleted for GCN2 in both RPE and HeLa cell lines (Fig. 6E, Fig S6B, C).

Together, these findings suggest that GCN2 influences cytoskeletal organization and actomyosin contractility. To gain insight into the underlying processes, we performed two independent exploratory proteomic screens (each run once); BioID proximity labelling with GCN2, and examined the PP1γ interactome upon GCN2 depletion (siGCN2 versus siCtr). We first curated two actin-related gene sets, the first containing the most canonical regulators of actin dynamics (*n* = 101) and an extended list (*n* = 256) (Table S5) encompassing broader adhesion junction components, based on established GO terms.

Among ca. 4500 quantified BioID hits, 70/101 and 127/256 overlapped with the curated gene sets, 10 and 19 of which were enriched > 2-fold over ligase control (Fig. 6F, Tables S5, S6), suggesting proximity to sites of actin assembly and turnover.

Quantitative PP1γ IP-MS revealed substantial changes in the set of actin regulatory proteins associated with PP1γ upon GCN2 depletion (Fig. 6G, Tables S5, S6). Components of the Arp2/3 machinery (ARPC1B, ARPC3, ACTR3), RhoA regulators (ROCK2, ARHGEF2), the RAC GAP SH3BP1, the formin DIAPH3, and adhesion proteins (BCAR1, CTNNA1/2, SDC4, MYO1C, FLNB, PALLD, MYH9 and PLEC) were enriched in PP1γ immunoprecipitates from siGCN2-transfected cells at least 3-fold. In addition, the WAVE regulator NCKAP1, the formin INF2, actin crosslinker FLNA, severing protein DSTN, capping proteins CAPZA1/2, CAPZB and LIM adaptors (TRIP6, AJUBA) were enriched at least 2-fold. In contrast, the WAVE scaffold ABI2, the integrin α4 ITGA4 and the actin severing protein CFL1 levels were either not detectable in samples depleted for GCN2 or reduced. Collectively, these results indicate that depletion of GCN2 alters the association of multiple actin regulatory proteins with PP1γ, including factors involved in actin nucleation, filament elongation, and adhesion-linked cytoskeletal organization. This broad distribution across different functions suggests that GCN2 perturbation impacts multiple components of the actin regulatory network, which likely contributes to the impaired migration observed upon GCN2 depletion or inhibition.

## Discussion

GCN2 has long been seen as an attractive target in the context of cancer. High levels of GCN2 in several cancers correlate with poor prognosis, as apparent in the depmap pan-cancer database [55, 56], as well as a number of previous studies. For example, Ye et al. [21] observed increases in both the total amount of GCN2 and phosphorylated GCN2 in colon, lung, breast and liver cancer tissue samples when compared with healthy tissue. Wang et al. [20] made similar observations in human oral squamous cell carcinoma, and, most recently, Furnish et al. [75] reported similar results in a prostate-cancer study. In addition, increased GCN2 expression levels in human papillary renal cell carcinoma were a powerful indicator of poor patient outcomes [76]. Furthermore, a recent study using the TCGA data revealed that GCN2 expression is elevated in 26 out of 29 cancer types [77]. However, elevated GCN2 expression levels do not always correlate with poor survival [55, 77]. Since exposure to stress is a general feature of cancers, this observation is difficult to interpret in the context of the canonical role in stress responses alone, indicating that additional functions also have to be considered.

In the current study, we performed correlation analyses of mRNA levels of GCN2 and that of other genes in patient samples from cervical cancer and found correlations not only with stress-response genes, but also with genes involved in mitosis and cell migration. Our functional analyses confirmed that these correlations indeed reveal novel functions. Furthermore, our analyses of growth benefits associated with elevated GCN2 levels suggest that the novel functions can contribute to aggressive disease in cancers with high GCN2 levels.

The mitotic role suggested from the analysis of patient data was confirmed in several cervical cancer cell lines. While the phenotype was not exactly the same in different cell lines, a common theme was that GCN2 inhibition severely interferes with mitotic progression in each of the cervical cancer cell lines we tested, as previously reported for cell lines derived from other types of cancer [37]. These data further support our previously published findings [37] and highlight the relevance of GCN2’s mitotic role for cancer. Furthermore, we have shown that cells engineered to overexpress GCN2 were resistant to some forms of mitotic stress. This might be an important factor contributing to the poor prognosis of patients with cancers with high levels of GCN2. Cancer cells often undergo tetraploidization and up to 70% of cancers are aneuploid, presumably providing growth advantages to the cancer cells [78–82]. However, in such cells aberrant and stressed mitoses are prevalent, leading to further genomic and chromosome abnormalities and further aberrant mitoses. In such a vicious circle even a small protection, as observed in cell lines with high GCN2 levels and exposed to drugs inducing mitotic stress in this study, might be beneficial for cancer cells. Furthermore, antimitotic agents are broadly used in chemotherapy and even if the protection by high levels of GCN2 in cell lines is modest, elevated GCN2 levels could contribute to resistance and should be considered when choosing therapeutic agents targeting mitosis.

The role in cell migration was confirmed in non-transformed epithelial cells, as well as two different cervical-cancer-derived cell lines. Previous studies implicated GCN2 in wound healing in keratinocytes [71] and poor wound healing in diabetes was associated with downregulation of GCN2 activity [65]. These studies attribute the involvement in wound healing to the canonical function through eIF2α phosphorylation. We previously identified PP1α and γ as novel GCN2 substrates in mitosis [37] and here investigated whether these substrates are relevant for cell migration. Several pieces of evidence suggest that eIF2α is not the only relevant substrate in the context of cell migration. First, in the present study GCN2 inhibition had a larger impact on cell migration than ISRIB, which counteracts eIF2α phosphorylation. Second, we previously showed that the two functions can be genetically separated by using a RAXA mutant, which is unable to bind PP1 but is able to phosphorylate eIF2α [37]. Here we found that the RAXA mutant was not able to rescue the migration defect after GCN2 knock-down. Third, the impact of ISRIB and the RAXA mutant on the wound-healing capacity was additive. Collectively, these data suggest that both substrates are important in the context of cell migration.

Actin reorganization during cell movement is regulated by extensive phosphorylation- dephosphorylation. While several kinases involved have been identified, the regulation of phosphatases is less well understood. Our exploratory proteomic analyses are consistent with a potential role for GCN2 in cytoskeletal regulation. Proximity labelling indicated that GCN2 localizes in the vicinity of numerous actin regulatory proteins, including components of the Arp2/3 nucleation machinery and regulators of actin filament turnover. In parallel, quantitative PP1γ interactome analysis pointed to GCN2-dependent changes in PP1γ-associated actin regulatory proteins, which span diverse functional categories. In light of the established role of GCN2 as a PP1γ regulator in mitosis [37], these observations raise the possibility that GCN2 modulates actin dynamics by influencing PP1-dependent phosphorylation networks. Future studies will be required to identify which actin regulators are directly controlled by this pathway and to define how these changes contribute to cytoskeletal remodelling during cell migration. Recently, noncanonical cytoskeletal functions have been reported for PERK, which scaffolds adhesion and actin-regulatory complexes during keratinocyte collective migration [83]. Our PP1γ interactome data indicate that GCN2 engages a distinct, PP1-dependent network of actin regulators. Together, these findings illustrate that kinases best known for phosphorylating eIF2α can adopt mechanistically distinct non-canonical roles at the cytoskeleton.

Strikingly, cells engineered to overexpress GCN2 displayed an increased ability to migrate towards a chemoattractant compared to isogenic control cells with “normal” GCN2 levels, which is apparent in wound healing, transwell and chemotaxis assays. Directional movement requires that cells can rearrange their cytoskeleton to move, as well as to respond to directional cues. A previous study suggested that the main reason for the impaired wound healing when GCN2 is inhibited is a diminished localized production of ROS in the leading edge of the wound [71]. Consistent with this model, in our chemotaxis assays GCN2 inhibition did impair directional movement, even if it did not lead to a complete loss of directionality (Fig. 4A). However, velocity was dramatically altered both in the presence of a GCN2 inhibitor and in cells overexpressing GCN2 (Fig. 4), suggesting that loss of ability to move is the primary reason for the impaired wound healing. One plausible explanation for this effect is that GCN2 impinges on cellular metabolism, which in turn affects cell migration. A recent work reported that GCN2 deficiency in diabetic patients enhanced oxidative phosphorylation, thereby promoting macrophage polarization, which in turn play important regulatory roles in inflammation and tissue repair during wound healing [65]. An additional plausible model emerging from our results is that GCN2 can also affect cell movement through modulating PP1 activity.

Cancer cells expressing high levels of GCN2 display faster migration and chemotactic ability, which might contribute to a higher potential to metastasize. Consistently, higher GCN2 levels correlate with higher metastatic potential in the MetMap database. An increased capacity to metastasize could be an important factor determining poor prognosis for patients with cancers with elevated GCN2 levels.

The analysis of correlations in the patient data revealed important new functions in mitosis and cell migration, which are highly relevant for cancer progression and can at least in part explain the poor prognosis associated with high GCN2 levels in many cancers. These roles could make GCN2 a valuable target for cancer therapies, especially in cancers with high GCN2 expression and poor prognosis. The novel results presented here will aid in better understanding of the impact of interfering with GCN2 activity and in exploiting it in therapy.

## Supplementary Information

Below is the link to the electronic supplementary material.

Supplementary Material 1

Supplementary Material 2

Supplementary Material 3

Supplementary Material 4

Supplementary Material 5

Supplementary Material 6

Supplementary Material 7

Supplementary Material 8

Supplementary Material 9

Supplementary Material 10

Supplementary Material 11

Supplementary Material 12

Supplementary Material 13

Supplementary Material 14

Supplementary Material 15

Supplementary Material 16

## Data Availability

The original contributions presented in this study are included in the article/supplementary material. Further inquiries can be directed to the corresponding author. Patient characteristics, treatment, tumor biopsies and data acquisition have been described previously [38, 39].

## References

[1] R.C. Wek, Role of eIF2alpha kinases in translational control and adaptation to cellular stress. Cold. Spring. Harb. Perspect. Biol. 2018, **10**(7) 10.1101/cshperspect.a032870 PMC6028073 29440070

[2] R.C. Wek, H.Y. Jiang, T.G. Anthony, Coping with stress: eIF2 kinases and translational control. Biochem. Soc. Trans. **34**(Pt 1), 7–11 (2006) 16246168 10.1042/BST20060007

[3] K. Pakos-Zebrucka, I. Koryga, K. Mnich, M. Ljujic, A. Samali, A.M. Gorman, The integrated stress response. EMBO Rep. **17**(10), 1374–1395 (2016) 27629041 10.15252/embr.201642195 PMC5048378

[4] H.P. Harding, Y. Zhang, H. Zeng, I. Novoa, P.D. Lu, M. Calfon, N. Sadri, C. Yun, B. Popko, R. Paules et al., An integrated stress response regulates amino acid metabolism and resistance to oxidative stress. Mol. Cell. **11**(3), 619–633 (2003) 12667446 10.1016/s1097-2765(03)00105-9

[5] P.D. Lu, H.P. Harding, D. Ron, Translation reinitiation at alternative open reading frames regulates gene expression in an integrated stress response. J. Cell. Biol. **167**(1), 27–33 (2004) 15479734 10.1083/jcb.200408003 PMC2172506

[6] Novoa I, Zeng H, Harding HP, Ron D: Feedback inhibition of the unfolded protein response by *GADD34*-mediated dephosphorylation of eIF2α. J. Cell Biol. **153**(5):1011 (2001) 10.1083/jcb.153.5.1011 PMC2174339 11381086

[7] I. Novoa, Y. Zhang, H. Zeng, R. Jungreis, H.P. Harding, D. Ron, Stress-induced gene expression requires programmed recovery from translational repression. EMBO J. **22**(5), 1180–1187 (2003) 12606582 10.1093/emboj/cdg112 PMC150345

[8] M. Costa-Mattioli, P. Walter, The integrated stress response: from mechanism to disease. Science. **368**(6489) (2020) 10.1126/science.aat5314 PMC8997189 32327570

[9] D.J. McConkey, The integrated stress response and proteotoxicity in cancer therapy. Biochem. Biophys. Res. Commun. **482**(3), 450–453 (2017) 28212730 10.1016/j.bbrc.2016.11.047 PMC5319732

[10] X. Tian, S. Zhang, L. Zhou, A.A. Seyhan, L. Hernandez Borrero, Y. Zhang, W.S. El-Deiry, Targeting the Integrated Stress Response in Cancer Therapy. Front. Pharmacol. **12**, 747837 (2021) 34630117 10.3389/fphar.2021.747837 PMC8498116

[11] F. Tameire, I.I. Verginadis, N.M. Leli, C. Polte, C.S. Conn, R. Ojha, C. Salas Salinas, F. Chinga, A.M. Monroy, W. Fu et al., ATF4 couples MYC-dependent translational activity to bioenergetic demands during tumour progression. Nat. Cell Biol. **21**(7), 889–899 (2019) 31263264 10.1038/s41556-019-0347-9 PMC6608727

[12] R. Vendramin, V. Katopodi, S. Cinque, A. Konnova, Z. Knezevic, S. Adnane, Y. Verheyden, P. Karras, E. Demesmaeker, F.M. Bosisio et al., Activation of the integrated stress response confers vulnerability to mitoribosome-targeting antibiotics in melanoma. J. Exp. Med. **218**(9) (2021) 10.1084/jem.20210571 PMC8424468 34287642

[13] L. Sanchez-Burgos, B. Navarro-González, S. García-Martín, O. Sirozh, J. Mota-Pino, E. Fueyo-Marcos, H. Tejero, M.E. Antón, M. Murga, F. Al-Shahrour et al., Activation of the integrated stress response is a vulnerability for multidrug-resistant FBXW7-deficient cells. EMBO Mol. Med. **14**(9), e15855 (2022) 35861150 10.15252/emmm.202215855 PMC9449593

[14] W. Dudka, G. Hoser, S.S. Mondal, L. Turos-Korgul, J. Swatler, M. Kusio-Kobialka, M. Wołczyk, A. Klejman, M. Brewinska-Olchowik, A. Kominek et al., Targeting integrated stress response with ISRIB combined with imatinib treatment attenuates RAS/RAF/MAPK and STAT5 signaling and eradicates chronic myeloid leukemia cells. BMC cancer. **22**(1), 1254 (2022) 36460969 10.1186/s12885-022-10289-w PMC9719211

[15] H.G. Nguyen, C.S. Conn, Y. Kye, L. Xue, C.M. Forester, J.E. Cowan, A.C. Hsieh, J.T. Cunningham, C. Truillet, F. Tameire et al., Development of a stress response therapy targeting aggressive prostate cancer. Sci. Transl. Med. **10**(439), eaar2036 (2018) 29720449 10.1126/scitranslmed.aar2036 PMC6045425

[16] L.T. Gold, G.R. Masson, GCN2: roles in tumour development and progression. Biochem. Soc. Trans. **50**(2), 737–745 (2022) 35311890 10.1042/BST20211252 PMC9162460

[17] S. Anda, R. Zach, B. Grallert, Activation of Gcn2 in response to different stresses. PLoS One. **12**(8), e0182143 (2017) 28771613 10.1371/journal.pone.0182143 PMC5542535

[18] B.A. Castilho, R. Shanmugam, R.C. Silva, R. Ramesh, B.M. Himme, E. Sattlegger, Keeping the eIF2 alpha kinase Gcn2 in check. Biochim. Biophys. Acta. **1843**(9), 1948–1968 (2014) 24732012 10.1016/j.bbamcr.2014.04.006

[19] I. Martinez-Reyes, M. Sanchez-Arago, J.M. Cuezva, AMPK and GCN2-ATF4 signal the repression of mitochondria in colon cancer cells. Biochem. J. **444**(2), 249–259 (2012) 22435535 10.1042/BJ20111829

[20] Y. Wang, Y. Ning, G.N. Alam, B.M. Jankowski, Z. Dong, J.E. Nör, P.J. Polverini, Amino acid deprivation promotes tumor angiogenesis through the GCN2/ATF4 pathway. Neoplasia. **15**(8), 989–997 (2013) 23908598 10.1593/neo.13262 PMC3730049

[21] J. Ye, M. Kumanova, L.S. Hart, K. Sloane, H. Zhang, De D.N. Panis, E. Bobrovnikova-Marjon, J.A. Diehl, D. Ron, C. Koumenis, The GCN2-ATF4 pathway is critical for tumour cell survival and proliferation in response to nutrient deprivation. EMBO J. **29**(12), 2082–2096 (2010) 20473272 10.1038/emboj.2010.81 PMC2892366

[22] R.A. Cordova, J. Misra, P.H. Amin, A.J. Klunk, N.P. Damayanti, K.R. Carlson, A.J. Elmendorf, H.-G. Kim, E.T. Mirek, B.D. Elzey et al., GCN2 eIF2 kinase promotes prostate cancer by maintaining amino acid homeostasis. eLife. **11**, e81083 (2022) 36107759 10.7554/eLife.81083 PMC9578714

[23] M.J. Halaby, K. Hezaveh, S. Lamorte, M.T. Ciudad, A. Kloetgen, B.L. MacLeod, M. Guo, A. Chakravarthy, T.D.S. Medina, S. Ugel et al., GCN2 drives macrophage and MDSC function and immunosuppression in the tumor microenvironment. Sci. Immunol. **4**(42), eaax8189 (2019) 31836669 10.1126/sciimmunol.aax8189 PMC7201901

[24] A. Rashidi, J. Miska, C. Lee-Chang, D. Kanojia, W.K. Panek, A. Lopez-Rosas, P. Zhang, Y. Han, T. Xiao, K.C. Pituch et al., GCN2 is essential for CD8(+) T cell survival and function in murine models of malignant glioma. Cancer Immunol. immunotherapy: CII. **69**(1), 81–94 (2020) 31844909 10.1007/s00262-019-02441-6 PMC6952559

[25] M. St Paul, S.D. Saibil, M. Kates, S. Han, S.C. Lien, R.C. Laister, K. Hezaveh, A. Kloetgen, S. Penny, T. Guo et al., Ex vivo activation of the GCN2 pathway metabolically reprograms T cells, leading to enhanced adoptive cell therapy. Cell. Rep. Med. 101465 (2024) 10.1016/j.xcrm.2024.101465 PMC10983112 38460518

[26] A.P. Drainas, W.-H. Hsu, A.E. Dallas, C.D. Poltorack, J.W. Kim, A. He, G.L. Coles, M. Baron, M.C. Bassik, J. Sage, GCN2 is a determinant of the response to WEE1 kinase inhibition in small-cell lung cancer. Cell. Rep. **43**(8), 114606 (2024) 39120974 10.1016/j.celrep.2024.114606 PMC11407228

[27] C.P. Tang, O. Clark, J.R. Ferrarone, C. Campos, A.S. Lalani, J.D. Chodera, A.M. Intlekofer, O. Elemento, I.K. Mellinghoff, GCN2 kinase activation by ATP-competitive kinase inhibitors. Nat. Chem. Biol. **18**(2), 207–215 (2022) 34949839 10.1038/s41589-021-00947-8 PMC9549920

[28] M. Szaruga, D.A. Janssen, de C. Miguel, G. Hodgson, A. Fatalska, A.P. Pitera, A. Andreeva, A. Bertolotti, Activation of the integrated stress response by inhibitors of its kinases. Nat. Commun. **14**(1), 5535 (2023) 37684277 10.1038/s41467-023-40823-8 PMC10491595

[29] A. Tsherniak, F. Vazquez, P.G. Montgomery, B.A. Weir, G. Kryukov, G.S. Cowley, S. Gill, W.F. Harrington, S. Pantel, J.M. Krill-Burger et al., Defining a Cancer Dependency Map. Cell. **170**(3), 564–576e516 (2017) 28753430 10.1016/j.cell.2017.06.010 PMC5667678

[30] P. Saavedra-García, M. Roman-Trufero, H.A. Al-Sadah, K. Blighe, E. López-Jiménez, M. Christoforou, L. Penfold, D. Capece, X. Xiong, Y. Miao et al., Systems level profiling of chemotherapy-induced stress resolution in cancer cells reveals druggable trade-offs. Proc. Natl. Acad. Sci. USA. **118**(17) (2021) 10.1073/pnas.2018229118 PMC8092411 33883278

[31] S.L. Lehman, S. Ryeom, C. Koumenis, Signaling through alternative Integrated Stress Response pathways compensates for GCN2 loss in a mouse model of soft tissue sarcoma. Sci. Rep. **5**, 11781 (2015) 26123823 10.1038/srep11781 PMC4485314

[32] M. Piecyk, M. Triki, P.A. Laval, C. Duret, J. Fauvre, L. Cussonneau, C. Machon, J. Guitton, N. Rama, B. Gibert et al., The stress sensor GCN2 differentially controls ribosome biogenesis in colon cancer according to the nutritional context. Mol. Oncol. (2023) 10.1002/1878-0261.13491 PMC11467793 37452637

[33] M. Román-Trufero, I.T. Kleijn, K. Blighe, J. Zhou, P. Saavedra-García, A. Gaffar, M. Christoforou, A. Bellotti, J. Abrahams, A. Atrih et al., An ISR-independent role of GCN2 prevents excessive ribosome biogenesis and mRNA translation. Life Sci. Alliance. **8**(5), e202403014 (2025) 40032489 10.26508/lsa.202403014 PMC11876863

[34] B. Grallert, E. Boye, The Gcn2 kinase as a cell cycle regulator. Cell. Cycle. **6**(22), 2768–2772 (2007) 17986863 10.4161/cc.6.22.4933

[35] T. Tvegård, H. Soltani, H.C. Skjølberg, M. Krohn, E.A. Nilssen, S.E. Kearsey, B. Grallert, E. Boye, A novel checkpoint mechanism regulating the G1/S transition. Genes Dev. **21**(6), 649–654 (2007) 17369398 10.1101/gad.421807 PMC1820939

[36] M. Menacho-Marquez, J. Perez-Valle, J. Arino, J. Gadea, J.R. Murguia, Gcn2p regulates a G1/S cell cycle checkpoint in response to DNA damage. Cell. Cycle. **6**(18), 2302–2305 (2007) 17890903 10.4161/cc.6.18.4668

[37] V. Stonyte, M. Mastrangelopoulou, R. Timmer, L. Lindbergsengen, M. Vietri, C. Campsteijn, B. Grallert, The GCN2/eIF2αK stress kinase regulates PP1 to ensure mitotic fidelity. EMBO Rep. **24**(8), e56100 (2023) 37291955 10.15252/embr.202256100 PMC10398673

[38] C.S. Fjeldbo, C.H. Julin, M. Lando, M.F. Forsberg, E.K. Aarnes, J. Alsner, G.B. Kristensen, E. Malinen, H. Lyng, Integrative Analysis of DCE-MRI and Gene Expression Profiles in Construction of a Gene Classifier for Assessment of Hypoxia-Related Risk of Chemoradiotherapy Failure in Cervical Cancer. Clin. cancer research: official J. Am. Association Cancer Res. **22**(16), 4067–4076 (2016) 10.1158/1078-0432.CCR-15-2322 27012812

[39] C. Halle, E. Andersen, M. Lando, E.K. Aarnes, G. Hasvold, M. Holden, R.G. Syljuasen, K. Sundfor, G.B. Kristensen, R. Holm et al., Hypoxia-induced gene expression in chemoradioresistant cervical cancer revealed by dynamic contrast-enhanced MRI. Cancer Res. **72**(20), 5285–5295 (2012) 22890239 10.1158/0008-5472.CAN-12-1085

[40] B. Bachtiary, P.C. Boutros, M. Pintilie, W. Shi, C. Bastianutto, J.H. Li, J. Schwock, W. Zhang, L.Z. Penn, I. Jurisica et al., Gene expression profiling in cervical cancer: an exploration of intratumor heterogeneity. Clin. cancer research: official J. Am. Association Cancer Res. **12**(19), 5632–5640 (2006) 10.1158/1078-0432.CCR-06-0357 17020965

[41] A. Subramanian, P. Tamayo, V.K. Mootha, S. Mukherjee, B.L. Ebert, M.A. Gillette, A. Paulovich, S.L. Pomeroy, T.R. Golub, E.S. Lander et al., Gene set enrichment analysis: a knowledge-based approach for interpreting genome-wide expression profiles. Proc. Natl. Acad. Sci. U S A **102**(43), 15545–15550 (2005) 16199517 10.1073/pnas.0506580102 PMC1239896

[42] A. Liberzon, C. Birger, H. Thorvaldsdóttir, M. Ghandi, J.P. Mesirov, P. Tamayo, The Molecular Signatures Database (MSigDB) hallmark gene set collection. Cell. Syst. **1**(6), 417–425 (2015) 26771021 10.1016/j.cels.2015.12.004 PMC4707969

[43] R Core Team, *R: A Language and Environment for Statistical Computing*, ed. by Vienna (R Foundation for Statistical Computing, Austria, 2023)

[44] E. Campeau, V.E. Ruhl, F. Rodier, C.L. Smith, B.L. Rahmberg, J.O. Fuss, J. Campisi, P. Yaswen, P.K. Cooper, P.D. Kaufman, A versatile viral system for expression and depletion of proteins in mammalian cells. PLoS One. **4**(8), e6529 (2009) 19657394 10.1371/journal.pone.0006529 PMC2717805

[45] L. Kubitz, S. Bitsch, X. Zhao, K. Schmitt, L. Deweid, A. Roehrig, E.C. Barazzone, O. Valerius, H. Kolmar, J. Béthune, Engineering of ultraID, a compact and hyperactive enzyme for proximity-dependent biotinylation in living cells. Commun. Biol. **5** (2022) 10.1038/s42003-022-03604-5 PMC9253107 35788163

[46] P. Bieling, K. Rottner, From WRC to Arp2/3: Collective molecular mechanisms of branched actin network assembly. Curr. Opin. Cell Biol. **80**, 102156 (2023) 36868090 10.1016/j.ceb.2023.102156

[47] K. Campellone, N. Lebek, V. King, Branching out in different directions: Emerging cellular functions for the Arp2/3 complex and WASP-family actin nucleation factors. Eur. J. Cell Biol. **102**, 151301–151301 (2023) 36907023 10.1016/j.ejcb.2023.151301 PMC10330494

[48] M. Chesarone, B. Goode, Actin nucleation and elongation factors: mechanisms and interplay. Curr. Opin. Cell Biol. **21 1**, 28–37 (2009) 19168341 10.1016/j.ceb.2008.12.001 PMC2671392

[49] J. Faix, K. Rottner, Ena/VASP proteins in cell edge protrusion, migration and adhesion. J. Cell Sci. **135**, 6 (2022) 10.1242/jcs.259226 35285496

[50] B. Goode, J. Eskin, S. Shekhar, Mechanisms of actin disassembly and turnover. J. Cell Biol. **222** (2023) 10.1083/jcb.202309021 PMC10638096 37948068

[51] K. Rottner, T. Stradal, B. Chen, WAVE regulatory complex. Curr. Biol. **31** (2021) 10.1016/j.cub.2021.01.086 PMC8882368 34033782

[52] J. Rotty, C. Wu, J. Bear, New insights into the regulation and cellular functions of the ARP2/3 complex. Nat. Rev. Mol. Cell. Bio. **14**, 7–12 (2012) 23212475 10.1038/nrm3492

[53] R. Dominguez, S. Namgoong, 4.4 actin filament nucleation and elongation. 31–47 (2012)

[54] H. Wioland, B. Guichard, Y. Senju, S. Myram, P. Lappalainen, A. Jégou, G. Romet-Lemonne, ADF/Cofilin Accelerates Actin Dynamics by Severing Filaments and Promoting Their Depolymerization at Both Ends. Curr. Biol. **27**, 1956–1967 (2017) 28625781 10.1016/j.cub.2017.05.048 PMC5505867

[55] A. Lánczky, B. Győrffy, Web-Based Survival Analysis Tool Tailored for Medical Research (KMplot): Development and Implementation. J. Med. Internet. Res. **23**(7), e27633 (2021) 34309564 10.2196/27633 PMC8367126

[56] Á. Nagy, G. Munkácsy, B. Győrffy, Pancancer survival analysis of cancer hallmark genes. Sci. Rep. **11**(1), 6047 (2021) 33723286 10.1038/s41598-021-84787-5 PMC7961001

[57] S.J. Lee, M.J. Swanson, E. Sattlegger, Gcn1 contacts the small ribosomal protein Rps10, which is required for full activation of the protein kinase Gcn2. Biochem. J. **466**(3), 547–559 (2015) 25437641 10.1042/BJ20140782

[58] H. Kubota, K. Ota, H. Kubota, Y. Sakaki, K. Ota, T. Ito, Y. Sakaki, T. Ito, Budding Yeast GCN1 Binds the GI Domain to Activate the eIF2alpha Kinase GCN2. J. Biol. Chem. **276**(20), 17591–17596 (2001) 11350982 10.1074/jbc.M011793200

[59] E. Sattlegger, A.G. Hinnebusch, Separate domains in GCN1 for binding protein kinase GCN2 and ribosomes are required for GCN2 activation in amino acid-starved cells. Embo J. **19**(23), 6622–6633 (2000) 11101534 10.1093/emboj/19.23.6622 PMC305848

[60] E. Sattlegger, A.G. Hinnebusch, Polyribosome binding by GCN1 is required for full activation of eukaryotic translation initiation factor 2{alpha} kinase GCN2 during amino acid starvation. J. Biol. Chem. **280**(16), 16514–16521 (2005) 15722345 10.1074/jbc.M414566200

[61] C.C. Wu, A. Peterson, B. Zinshteyn, S. Regot, R. Green, Ribosome Collisions Trigger General Stress Responses to Regulate Cell Fate. Cell. **182**(2), 404–416 (2020). e414 32610081 10.1016/j.cell.2020.06.006 PMC7384957

[62] J. Han, S.H. Back, J. Hur, Y.-H. Lin, R. Gildersleeve, J. Shan, C.L. Yuan, D. Krokowski, S. Wang, M. Hatzoglou et al., ER-stress-induced transcriptional regulation increases protein synthesis leading to cell death. Nat. Cell Biol. **15**(5), 481–490 (2013) 23624402 10.1038/ncb2738 PMC3692270

[63] S. Schmidt, D. Gay, F.W. Uthe, S. Denk, M. Paauwe, N. Matthes, M.E. Diefenbacher, S. Bryson, F.C. Warrander, F. Erhard et al., A MYC-GCN2-eIF2α negative feedback loop limits protein synthesis to prevent MYC-dependent apoptosis in colorectal cancer. Nat. Cell. Biol. **21**(11), 1413–1424 (2019) 31685988 10.1038/s41556-019-0408-0 PMC6927814

[64] J.C. Ghosh, M. Perego, E. Agarwal, I. Bertolini, Y. Wang, A.R. Goldman, H.Y. Tang, A.V. Kossenkov, C.J. Libby, L.R. Languino et al., Ghost mitochondria drive metastasis through adaptive GCN2/Akt therapeutic vulnerability. Proc. Natl. Acad. Sci. USA. **119**(8) (2022) 10.1073/pnas.2115624119 PMC8872753 35177476

[65] Y. Hou, D. Wei, Z. Zhang, T. Lei, S. Li, J. Bao, H. Guo, L. Tan, X. Xie, Y. Zhuang et al., Downregulation of nutrition sensor GCN2 in macrophages contributes to poor wound healing in diabetes. Cell. Rep. **43**(1), 113658 (2024) 38175755 10.1016/j.celrep.2023.113658

[66] M. Keil, J.K. Sonner, T.V. Lanz, I. Oezen, T. Bunse, S. Bittner, H.V. Meyer, S.G. Meuth, W. Wick, M. Platten, General control non-derepressible 2 (GCN2) in T cells controls disease progression of autoimmune neuroinflammation. J. Neuroimmunol. **297**, 117–126 (2016) 27397084 10.1016/j.jneuroim.2016.05.014

[67] H. Orsini, L.P. Araujo, J.T. Maricato, M.G. Guereschi, M. Mariano, B.A. Castilho, A.S. Basso, GCN2 kinase plays an important role triggering the remission phase of experimental autoimmune encephalomyelitis (EAE) in mice. Brain. Behav. Immun. **37**, 177–186 (2014) 24362236 10.1016/j.bbi.2013.12.012

[68] B. Ravishankar, H. Liu, R. Shinde, K. Chaudhary, W. Xiao, J. Bradley, M. Koritzinsky, M.P. Madaio, T.L. McGaha, The amino acid sensor GCN2 inhibits inflammatory responses to apoptotic cells promoting tolerance and suppressing systemic autoimmunity. Proc. Natl. Acad. Sci. U S A **112**(34), 10774–10779 (2015) 26261340 10.1073/pnas.1504276112 PMC4553766

[69] I.R. Powley, A. Kondrashov, L.A. Young, H.C. Dobbyn, K. Hill, I.G. Cannell, M. Stoneley, Y.W. Kong, J.A. Cotes, G.C. Smith et al., Translational reprogramming following UVB irradiation is mediated by DNA-PKcs and allows selective recruitment to the polysomes of mRNAs encoding DNA repair enzymes. Genes Dev. **23**(10), 1207–1220 (2009) 19451221 10.1101/gad.516509 PMC2685536

[70] A. Luna, F. Elloumi, S. Varma, Y. Wang, N. Rajapakse Vinodh, M.I. Aladjem, J. Robert, C. Sander, Y. Pommier, W.C. Reinhold, CellMiner Cross-Database (CellMinerCDB) version 1.2: Exploration of patient-derived cancer cell line pharmacogenomics. Nucleic Acids Res. **49**(D1), D1083–D1093 (2020) 10.1093/nar/gkaa968 PMC7779001 33196823

[71] R.R. Miles, P.H. Amin, M.B. Diaz, J. Misra, E. Aukerman, A. Das, N. Ghosh, T. Guith, M.D. Knierman, S. Roy et al., The eIF2 kinase GCN2 directs keratinocyte collective cell migration during wound healing via coordination of reactive oxygen species and amino acids. J. Biol. Chem. **297**(5), 101257 (2021) 34597669 10.1016/j.jbc.2021.101257 PMC8554533

[72] X. Jin, Z. Demere, K. Nair, A. Ali, G.B. Ferraro, T. Natoli, A. Deik, L. Petronio, A.A. Tang, C. Zhu et al., A metastasis map of human cancer cell lines. Nature. **588**(7837), 331–336 (2020) 33299191 10.1038/s41586-020-2969-2 PMC8439149

[73] A.F. Zyryanova, K. Kashiwagi, C. Rato, H.P. Harding, A. Crespillo-Casado, L.A. Perera, A. Sakamoto, M. Nishimoto, M. Yonemochi, M. Shirouzu et al., ISRIB Blunts the Integrated Stress Response by Allosterically Antagonising the Inhibitory Effect of Phosphorylated eIF2 on eIF2B. Mol. Cell. **81**(1), 88–103e106 (2021) 33220178 10.1016/j.molcel.2020.10.031 PMC7837216

[74] M. Gomes, S. Letzian, M. Saynisch, S. Iden, Immunohistochemistry for phospho-myosin light chain 2 in adult murine skin. (2019)

[75] M. Furnish, D.P. Boulton, V. Genther, D. Grofova, M.L. Ellinwood, L. Romero, M.S. Lucia, S.D. Cramer, M.C. Caino, MIRO2 regulates prostate cancer cell growth via GCN1-dependent stress signaling. Mol. Cancer Res. (2022) 10.1158/1541-7786.MCR-21-0374 PMC8983529 34992146

[76] L. Ge, W. Chen, W. Cao, G. Liu, Q. Zhang, J. Zhuang, M. Zhang, J. Yang, S. Guo, X. Zhao et al., GCN2 is a potential prognostic biomarker for human papillary renal cell carcinoma. Cancer Biomarkers. **22**, 395–403 (2018) 29865032 10.3233/CBM-170922 PMC13078478

[77] C. Chen, Y. Xie, S. Qian, Multifaceted role of GCN2 in tumor adaptation and therapeutic targeting. Transl Oncol. **49**, 102096 (2024) 39178574 10.1016/j.tranon.2024.102096 PMC11388189

[78] J.M. Sheltzer, A. Amon, The aneuploidy paradox: costs and benefits of an incorrect karyotype. Trends Genet. **27**(11), 446–453 (2011) 21872963 10.1016/j.tig.2011.07.003 PMC3197822

[79] S.D. Rutledge, T.A. Douglas, J.M. Nicholson, M. Vila-Casadesús, C.L. Kantzler, D. Wangsa, M. Barroso-Vilares, S.D. Kale, E. Logarinho, D. Cimini, Selective advantage of trisomic human cells cultured in non-standard conditions. Sci. Rep. **6**, 22828 (2016) 26956415 10.1038/srep22828 PMC4783771

[80] U. Ben-David, G. Arad, U. Weissbein, B. Mandefro, A. Maimon, T. Golan-Lev, K. Narwani, A.T. Clark, P.W. Andrews, N. Benvenisty et al., Aneuploidy induces profound changes in gene expression, proliferation and tumorigenicity of human pluripotent stem cells. Nat. Commun. **5**, 4825 (2014) 25198699 10.1038/ncomms5825

[81] N.K. Chunduri, Z. Storchová, The diverse consequences of aneuploidy. Nat. Cell Biol. **21**(1), 54–62 (2019) 30602769 10.1038/s41556-018-0243-8

[82] B.A. Weaver, D.W. Cleveland, The aneuploidy paradox in cell growth and tumorigenesis. Cancer Cell. **14**(6), 431–433 (2008) 19061834 10.1016/j.ccr.2008.11.011 PMC3132552

[83] M.B. Diaz, K.A. Staschke, A.J. 2 Baucum nd, D.F. Spandau, R.C. Wek, PERK protein kinase facilitates keratinocyte collective cell migration by engagement with cell adhesion molecules, independent of its kinase activity. Mol. Biol. Cell. **36**(11), ar40 (2025) 40991407 10.1091/mbc.E25-06-0277 PMC12562022

