# Supplementary material for: Elevated GCN2 levels in cancer cells confer protection from mitotic stress and faster cell movement
Source: Cell Oncol (Dordr). 2026 May 11;49(4):92. doi: 10.1007/s13402-026-01214-5 (PMC13346413; doi:10.1007/s13402-026-01214-5)
Supplement: Supplementary file 1 — Supplementary Material 1 [file 13402_2026_1214_MOESM1_ESM.docx]

# Supplementary Information

**File 1**, Overview and legends to movies

**Files 2-8** Supplementary movies

**File 9** Source files for immunoblots

**File 10** Supplementary figures with legends

**Files 11-16** Supplementary tables

# Movies

Movie S1 SiHa cell going through mitosis in the absence of GCN2i. Related to Fig S3. Images were taken every 5 min, tubulin is shown in magenta, DNA is shown in gray.

Movie S2 SiHa cells delay mitotic progression in the presence of 2 µM GCN2i. Related to Fig S3. Images were taken every 5 min, tubulin is shown in magenta, DNA is shown in gray.

Movie S3 Caski cell going through mitosis in the absence of GCN2i. Related to Fig S3. Images were taken every 5 min, tubulin is shown in magenta, DNA is shown in gray.

Movie S4 Caski cells delay mitotic progression in the presence of 2 µM GCN2i. Related to Fig S3. Images were taken every 5 min, tubulin is shown in magenta, DNA is shown in gray.

Movie S5 Wound healing in control cells. Related to Fig 4E. Images were taken every 30 min.

Movie S6 Wound healing in cells incubated in the presence of 2 µM GCN2i. Related to Fig 4E. Images were taken every 30 min.

Movie S7 Wound healing in cells stably overexpressing GCN2 (GCN2^high^). Related to Fig 4E. Images were taken every 30 min.
