# Supplementary material for: Elevated GCN2 levels in cancer cells confer protection from mitotic stress and faster cell movement
Source: Cell Oncol (Dordr). 2026 May 11;49(4):92. doi: 10.1007/s13402-026-01214-5 (PMC13346413; doi:10.1007/s13402-026-01214-5)
Supplement: Supplementary file 9 — Supplementary Material 9 [file 13402_2026_1214_MOESM9_ESM.pdf]

Source files for immunoblots

The file contains uncropped images of the immunoblots. Each figure panel showing immunoblots is shown on two pages.

The first page shows the blots, luminescence signals overlayed with colorimetric images. We cut our blots to reduce the number of strippings. It is described for each blot below how they were cut and probed, and the position of the size markers is shown.

Then chemiluminescence signals for each figure panel are shown with clipping masks released. The bands used in the figures are indicated by cyan rectangles (same rectangles as the black ones in the main figures).

Each blue rectangle frames one exposure for all the strips, including the one shown in the figure and also including several over- or underexposed bands of other bands corresponding to other proteins detected on the other strips.

Fig 5C is a composite of two different exposures; again the bands used in the main figure are indicated by cyan rectangles. Red bands indicate saturated exposures, please note that none of these were used in the figures.

Figure 3B

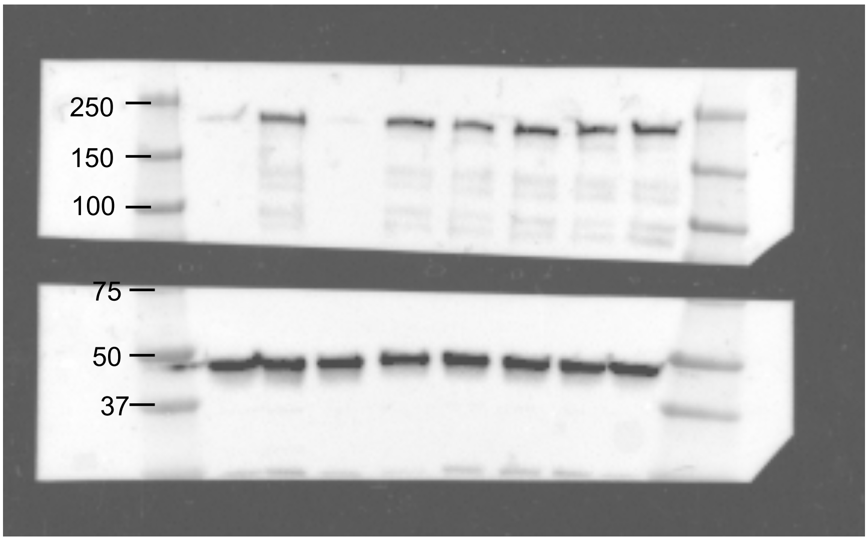

The blot was cut between the 100 and 75 kDa markers.
The upper part was probed with a GCN2 antibody.
The lower part was probed with a γ-tubulin antibody.

Figure panel GCN2

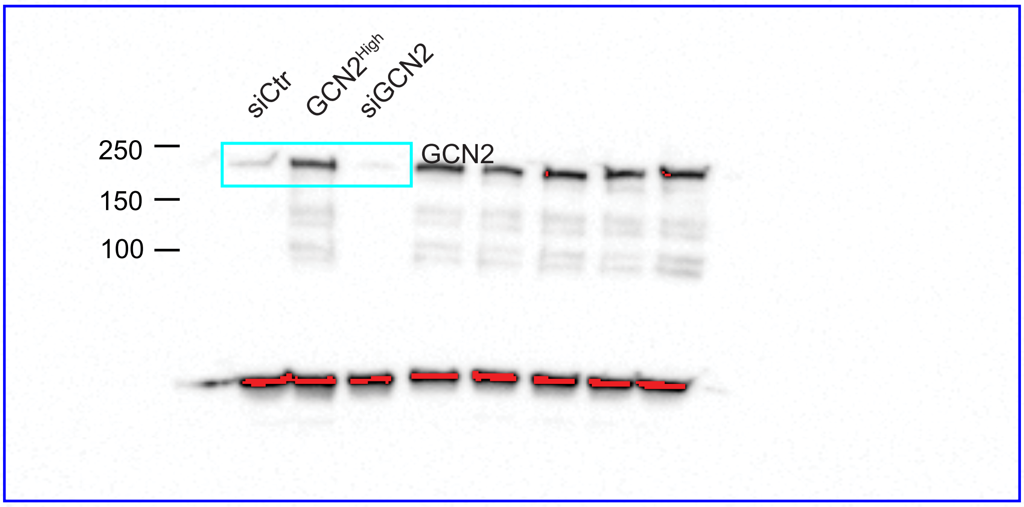

Figure panel γ-tubulin

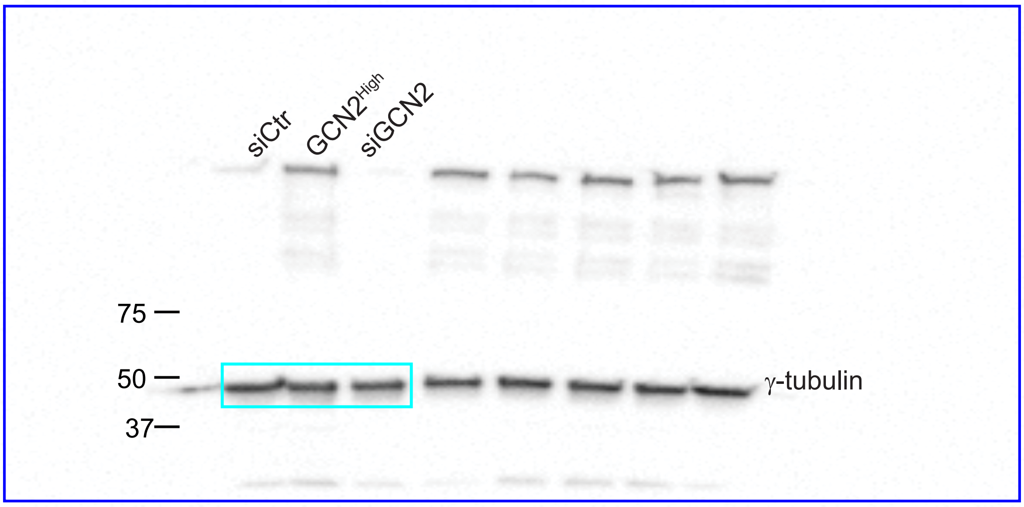

Figure 3 A Migration capacity correlates with GCN2 levels in hTert-RPE1 cells. hTert-RPE1 cells were transfected with GCN2-targeting siRNA for 48 h (siGCN2) or transduced to stably overexpress GCN2 (GCN2high). Cells were seeded without FBS into transwell chambers with 8 µM pore size and exposed to an FBS gradient for 16 hours. Migrated cells were counted after DAPI staining and normalized to seeding controls. Three independent experiments, mean and STDEV are shown.
B Immunoblots of lysates of cells from the experiment shown in A. γ-tubulin is used as a loading control.

Figure 5C

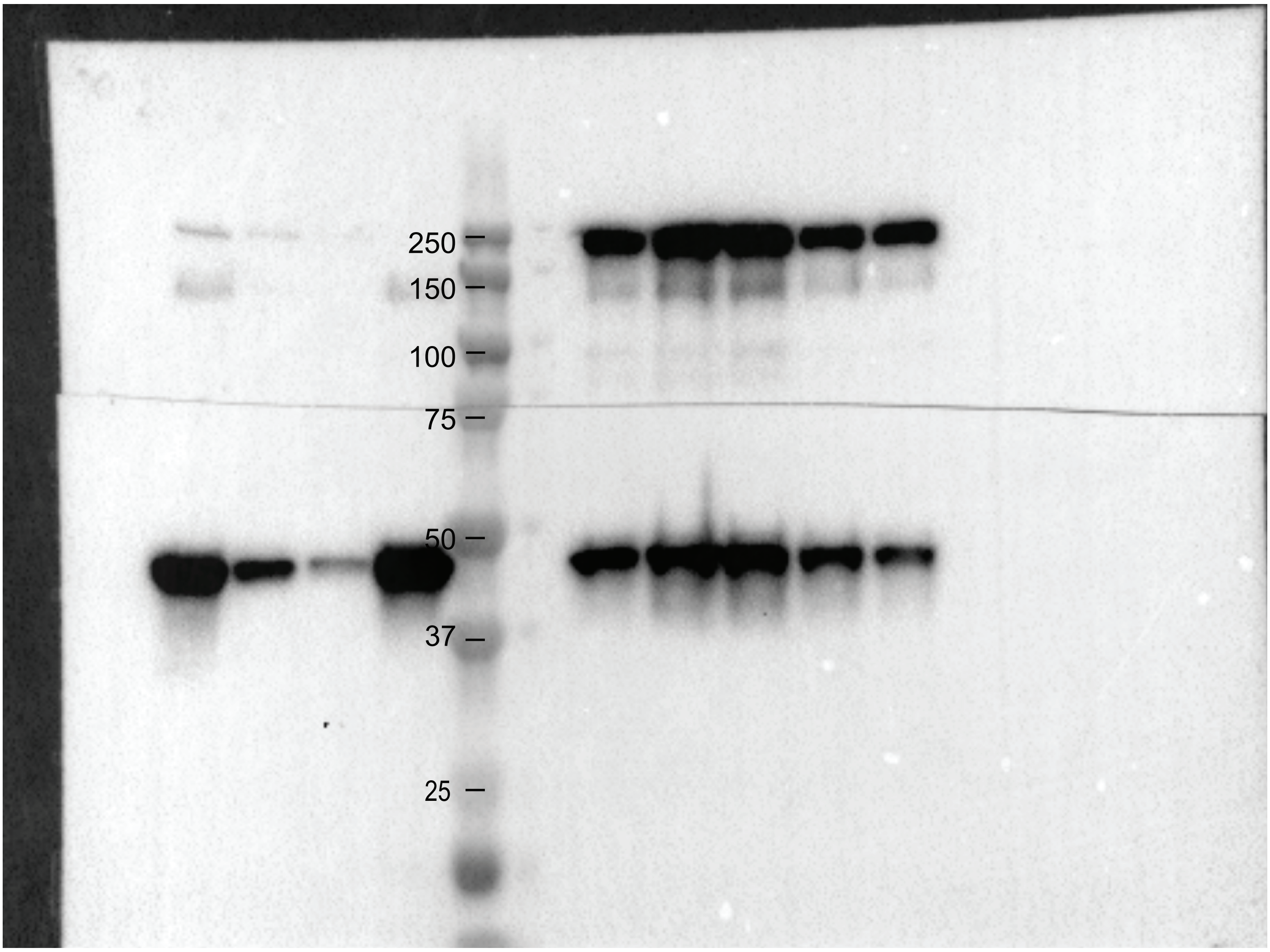

The blot was cut at the 75 kDa marker.
The upper part was probed with a GCN2 antibody.
The lower part was probed with a γ-tubulin antibody.
The bands shown in the figure are framed in black rectangles.

Long exposure, figure panel GCN2 non-transduced cells

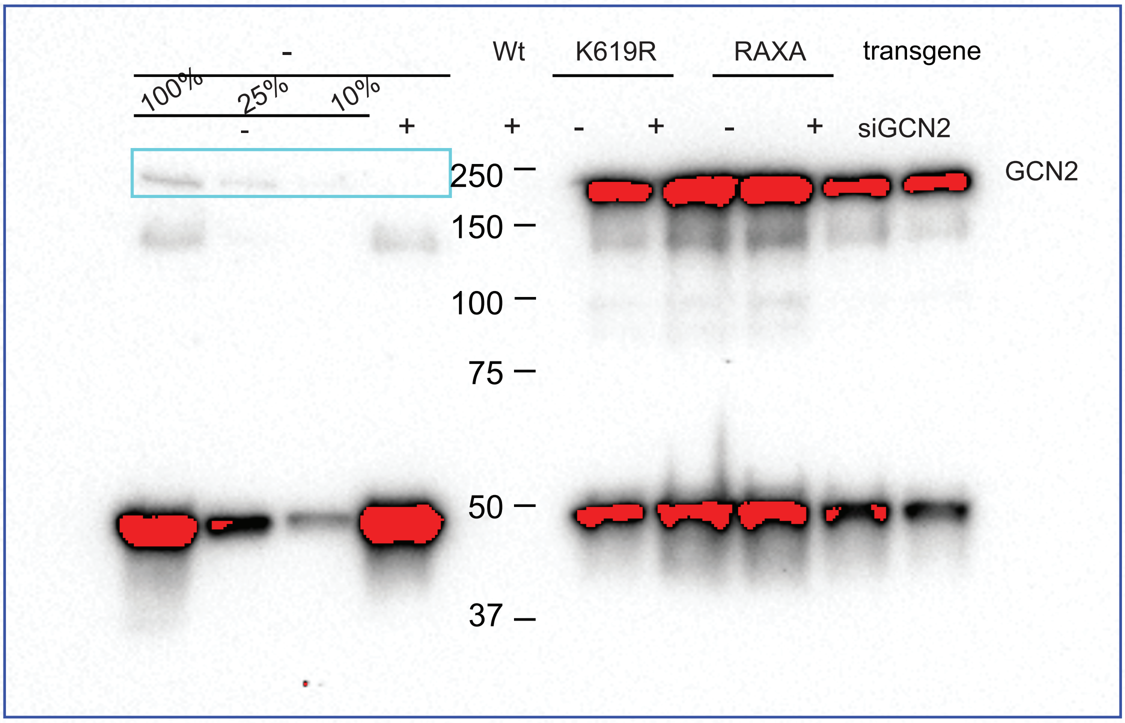

Short exposure, figure panel GCN2 in transduced cells and γ-tubulin

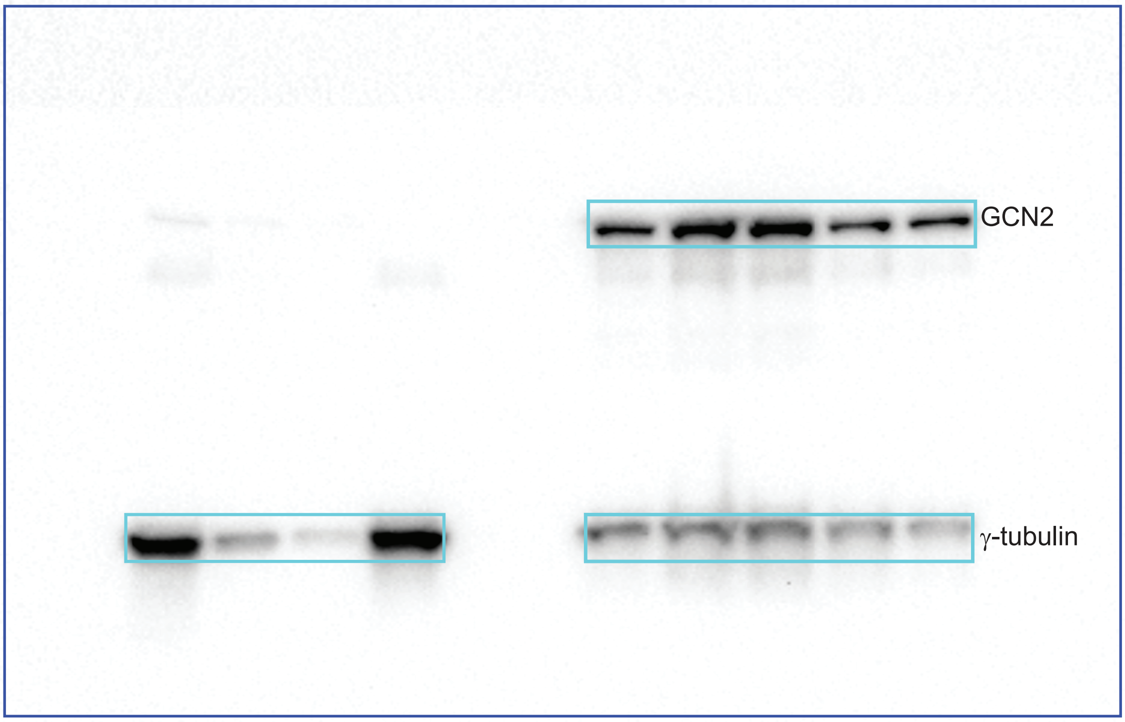

Figure 5C Knock-down efficiency and expression of the mutant transgenes in the experiments shown in C was tested by immunoblotting. Different exposures of the same blot are shown for GCN2. γ-tubulin is shown as loading control.

Figure S3A

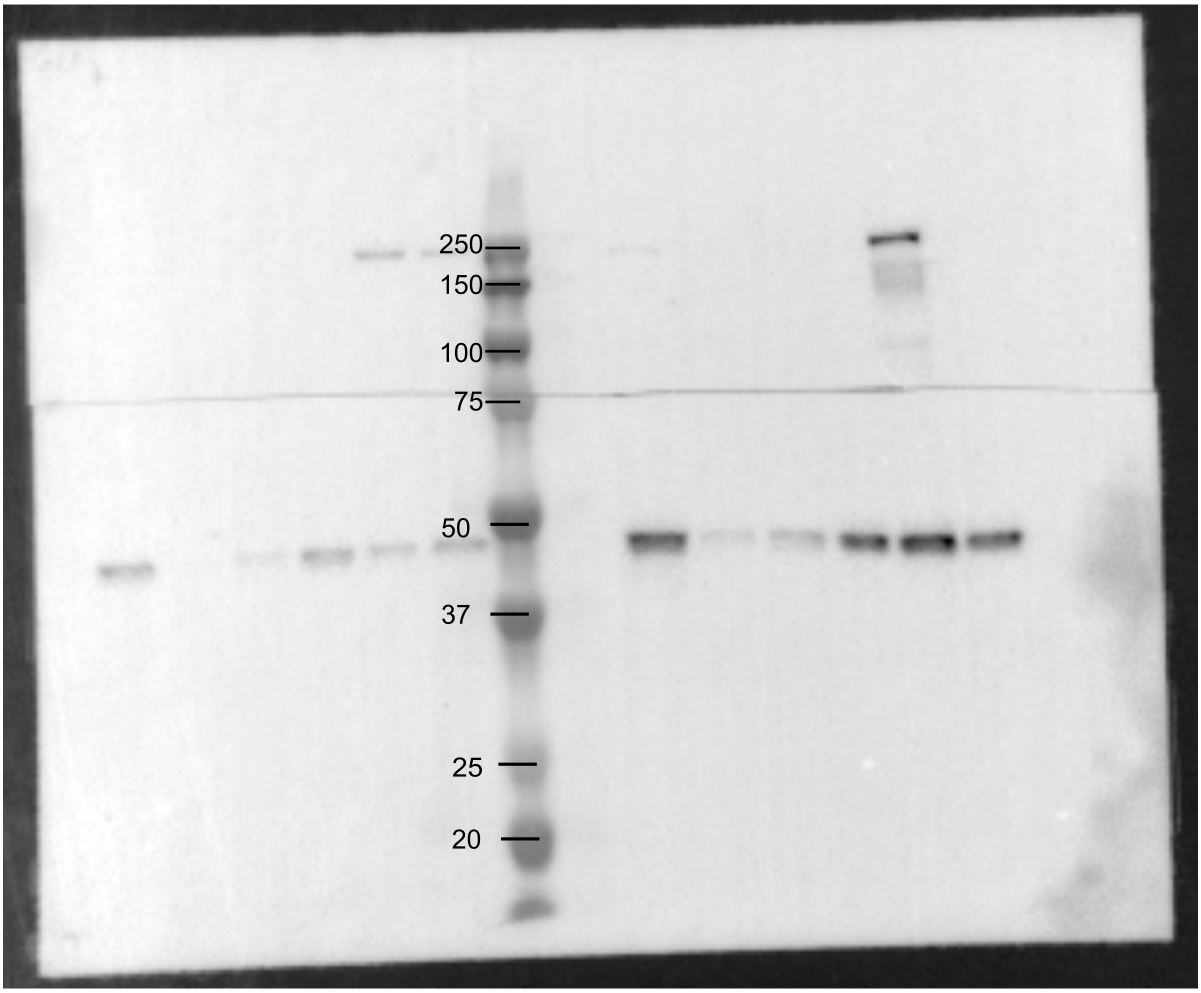

The blot was cut at 75 kDa.
The upper part was probed with a GCN2 antibody.
The lower part was probed with a γ-tubulin antibody.
Note that the GCN2 signal after knock-down was too weak after developing with the Pico Substrate (visible on the source data for the γ-tubulin panel and on the merged image), and was redeveloped using the femto substrate (shown in the figure).

Figure panel GCN2

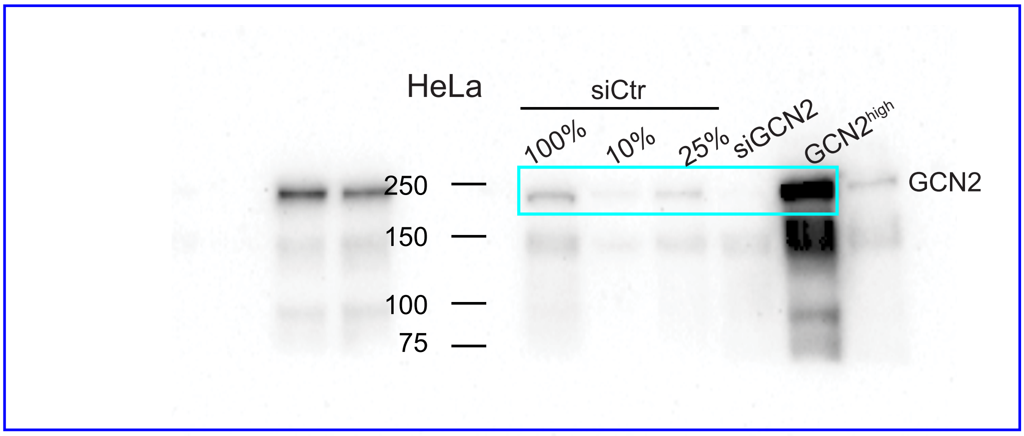

Figure panel γ-tubulin

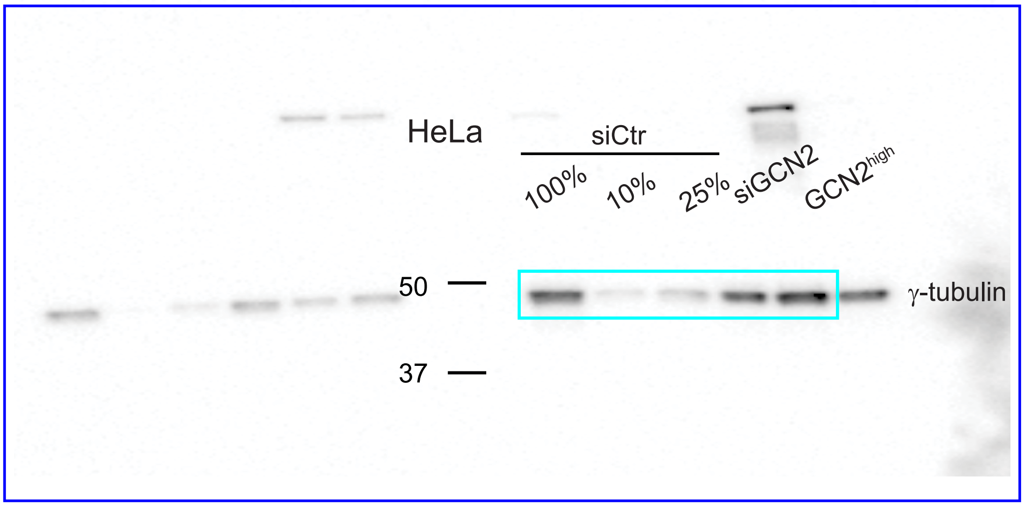

Figure S3 A, B Representative immunoblots to show GCN2 levels in (B) HeLa and (C) hTert-RPE1 cell lines. Cells were transfected with GCN2-targeting siRNA-s to deplete (siGCN2) or were engineered to stably overexpress GCN2 by lentiviral transduction (GCN2high). To estimate the efficiency of depletion, different amounts (100%, 10% and 25%; corresponds to 30, 3 and 7.5 μg protein, respectively) of the lysate from cells transfected with control siRNA was loaded, along with 30 μg each of the transfected and the overexpressing samples. γ-tubulin is shown as a loading control.

Figure S3 B

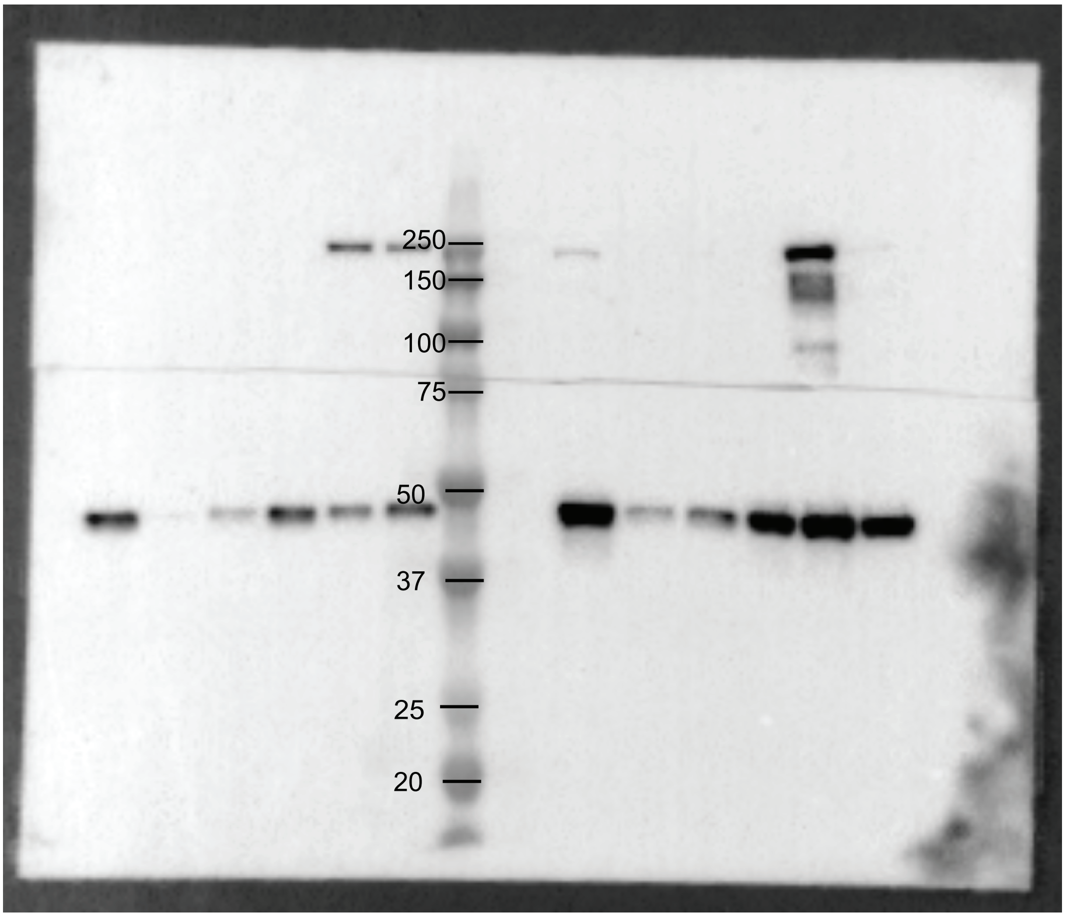

The blot was cut at 75 kDa.
The upper part was probed with a GCN2 antibody.
The lower part was probed with a γ-tubulin antibody.
Note that the GCN2 signal after knock-down was too weak after developing with the Pico Substrate (visible on the source data for the γ-tubulin panel and on the merged image), and was redeveloped using the femto substrate (shown in the figure).

Figure panel GCN2

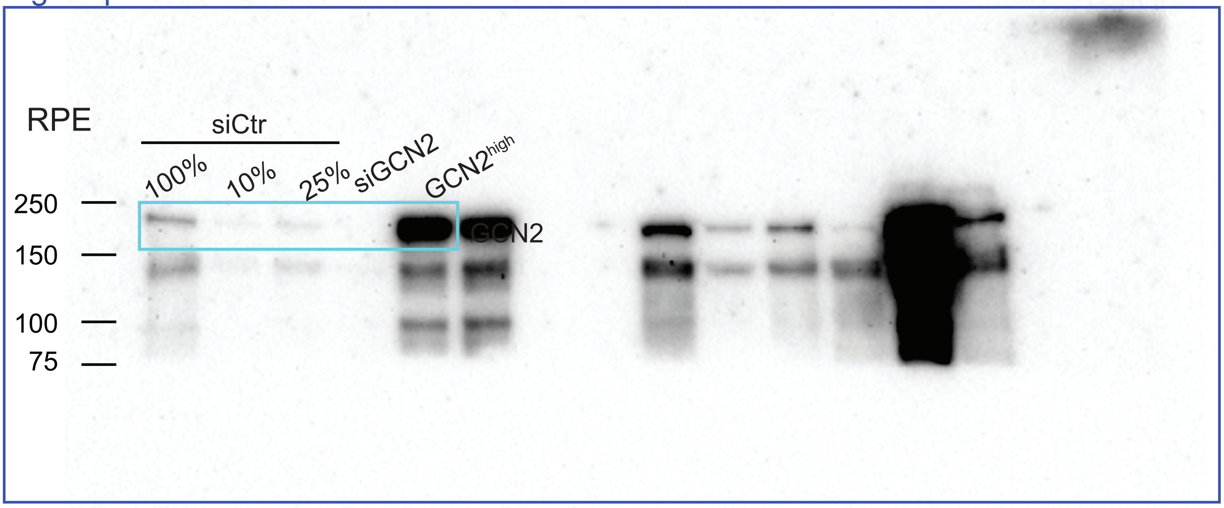

Figure panel γ-tubulin

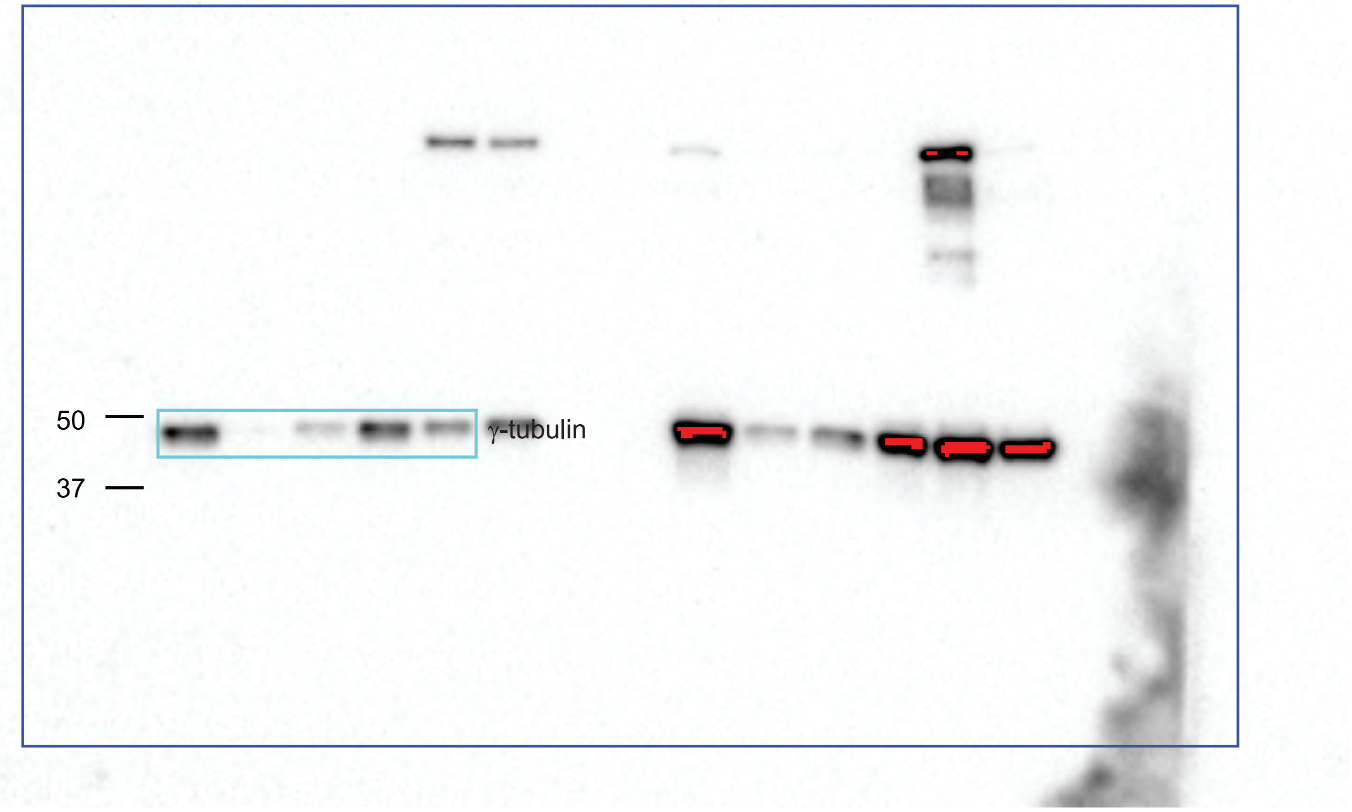

Figure S3 A, B Representative immunoblots to show GCN2 levels in (B) HeLa and (C) hTert-RPE1 cell lines. Cells were transfected with GCN2-targeting siRNA-s to deplete (siGCN2) or were engineered to stably overexpress GCN2 by lentiviral transduction (GCN2high). To estimate the efficiency of depletion, different amounts (100%, 10% and 25%; corresponds to 30, 3 and 7.5 μg protein, respectively) of the lysate from cells transfected with control siRNA was loaded, along with 30 μg each of the transfected and the overexpressing samples. γ-tubulin is shown as a loading control.

Figure S5 E

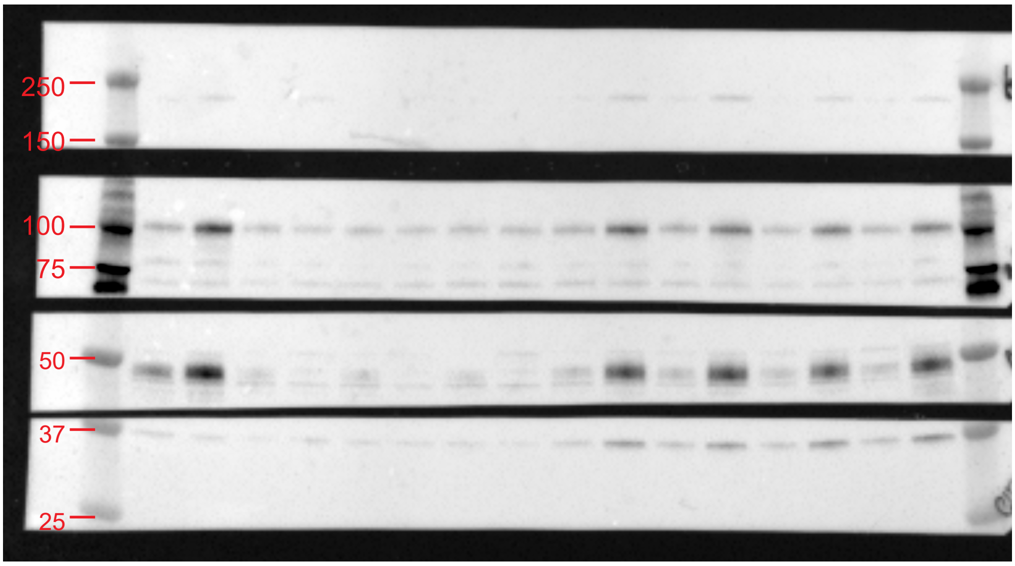

The blot was cut at the 150, below 75 and just above the 37 kDa markers. The upmost strip was probed with an antibody against GCN2-P, stripped and reproped for GCN2.
The 150-75 kDa strip was probed for GADD34.
The <50 kDa strip was probed with an antibody against eIF2α–P, stripped and reprobed for eIF2α. The 75-50 kDa strip was probed for γ-tubulin. The merged image above shows the marker bands and how the blot was cut. The images of the bands shown in the figure are shown below, with the clipping masks released.

Figure panel GCN2-P

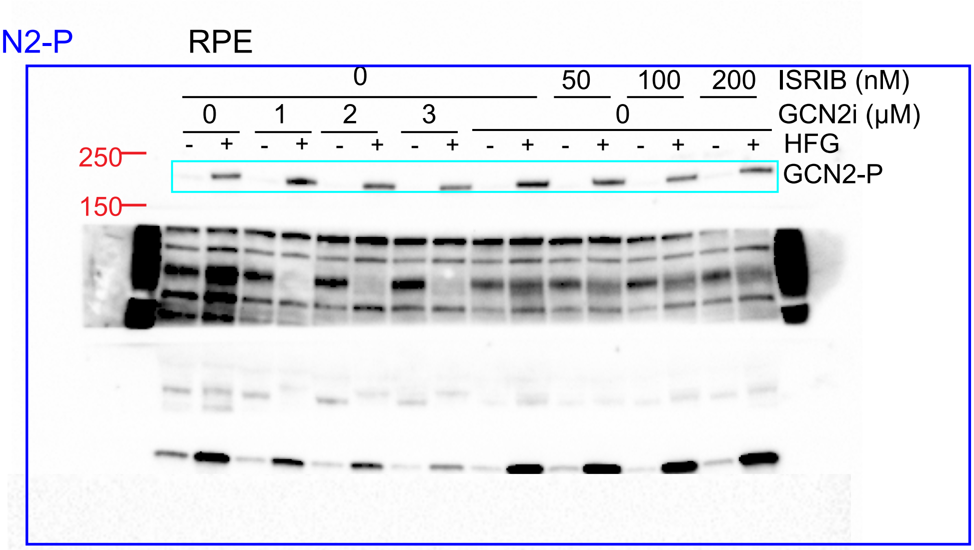

Figure panel GCN2

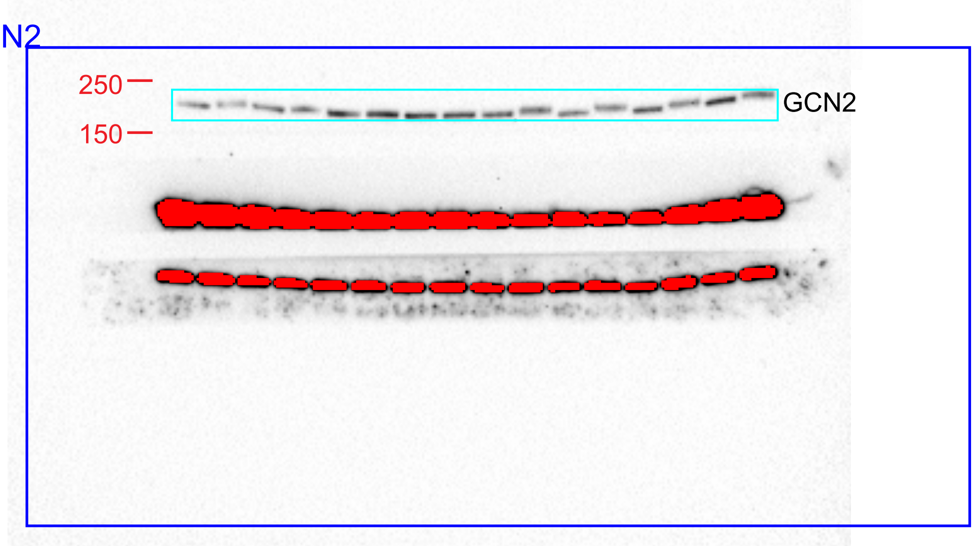

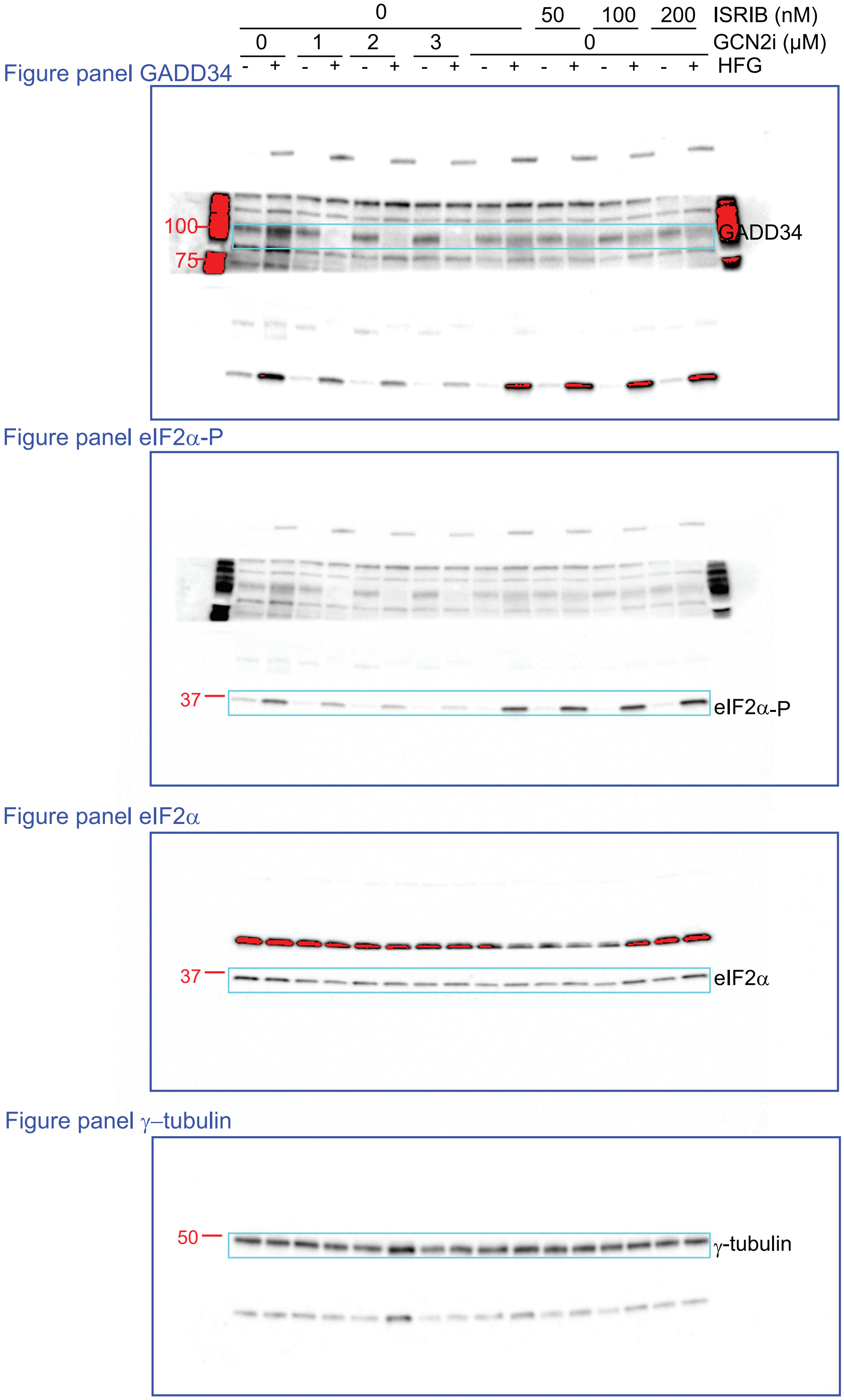

Figure S5 E, F hTert-RPE1(E) and HeLa (F) cells were treated with 600 nM (E) or 60 nM (F) HFG in the presence of ISRIB or GCN2i as indicated, and GCN2 autophosphorylation, GADD34 induction and eIF2α phosphorylation were assessed by immunoblotting. GAPDH and γ-tubulin are shown as loading control. Related to Fig 5A, B.

Figure S5 F

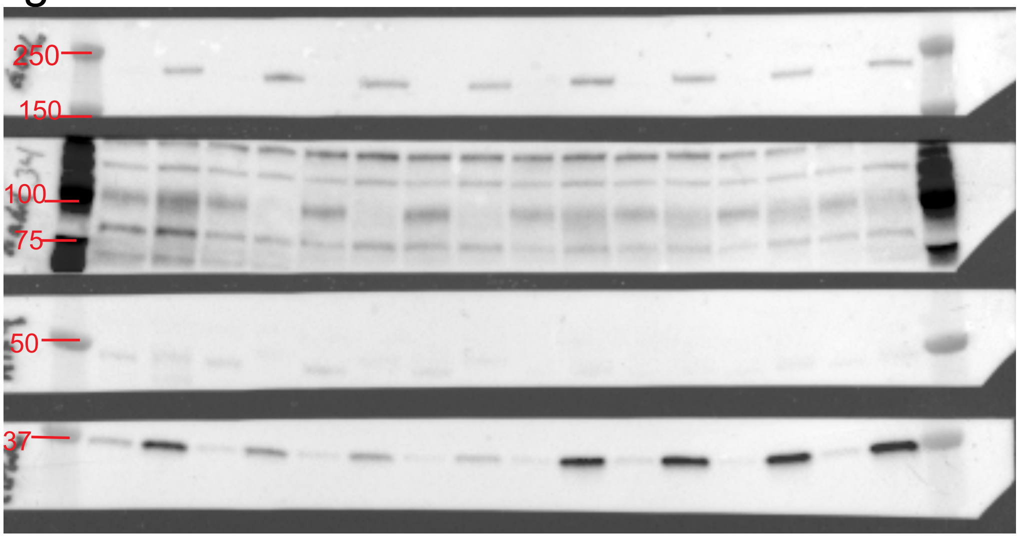

The blot was cut at the 150, below 75 and just above the 37 kDa markers. The upmost strip was probed with an antibody against GCN2-P, stripped and reproped for GCN2.
The 150-75 kDa strip was probed for GADD34.
The <50 kDa strip was probed with an antibody against eIF2α–P, stripped and reprobed for eIF2α. The 75-50 kDa strip was probed for γ-tubulin. The merged image above shows the marker bands and how the blot was cut. The images of the bands shown in the figure are shown below, with the clipping masks released. The bands shown in the figure are framed in black rectangles.

Figure panel GCN2-P

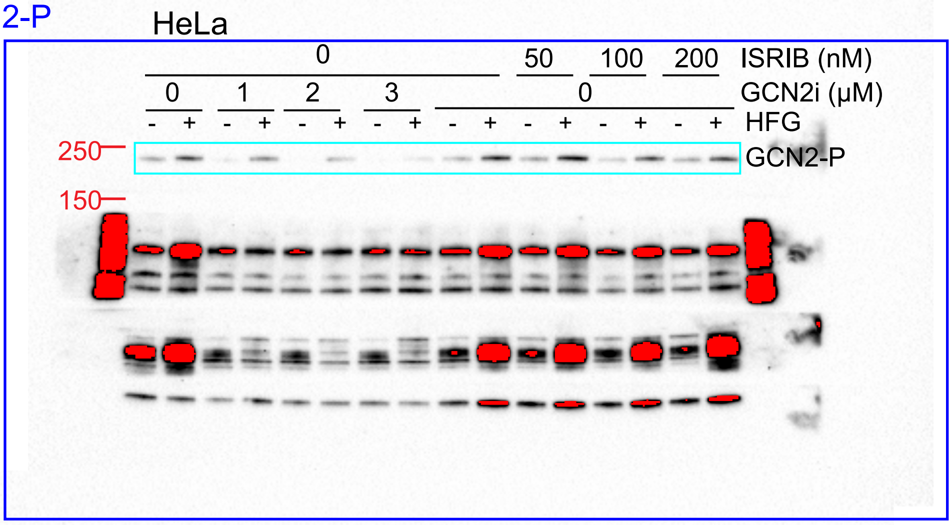

Figure panel GCN2

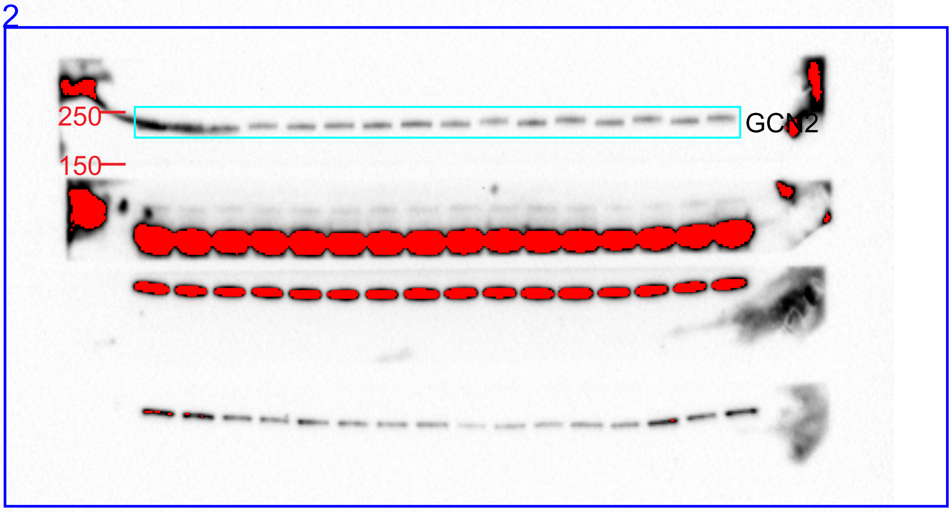

Figure S5 E

Figure panel GADD34

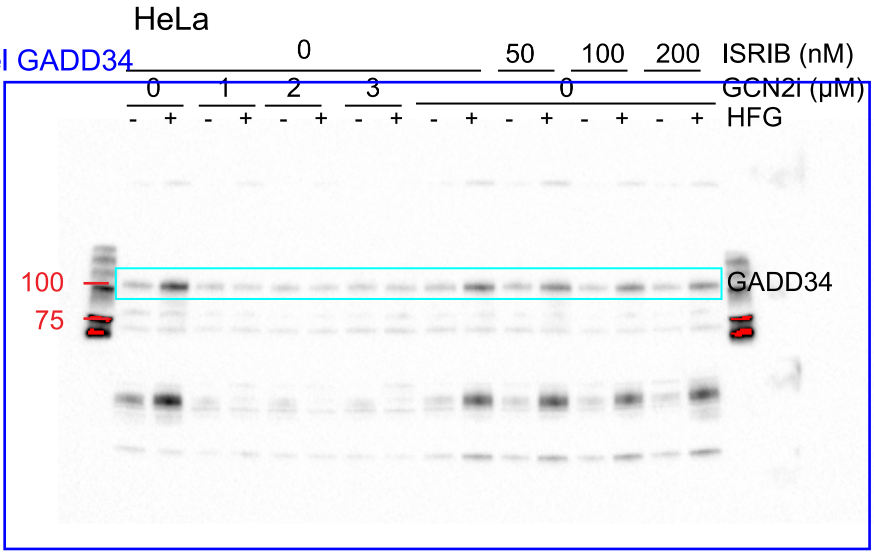

Figure panel eIF2α-P

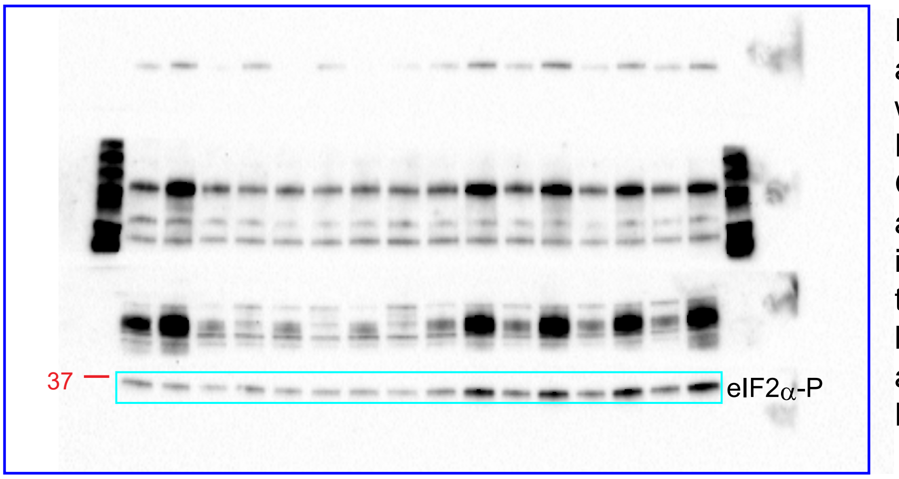

Figure S5 E, F hTert-RPE1(E) and HeLa (F) cells were treated with 600 nM (E) or 60 nM (F) HFG in the presence of ISRIB or GCN2i as indicated, and GCN2 autophosphorylation, GADD34 induction and eIF2α phosphorylation were assessed by immunoblotting. GAPDH and γ-tubulin are shown as loading control. Related to Fig 5A, B.

Figure panel eIF2α

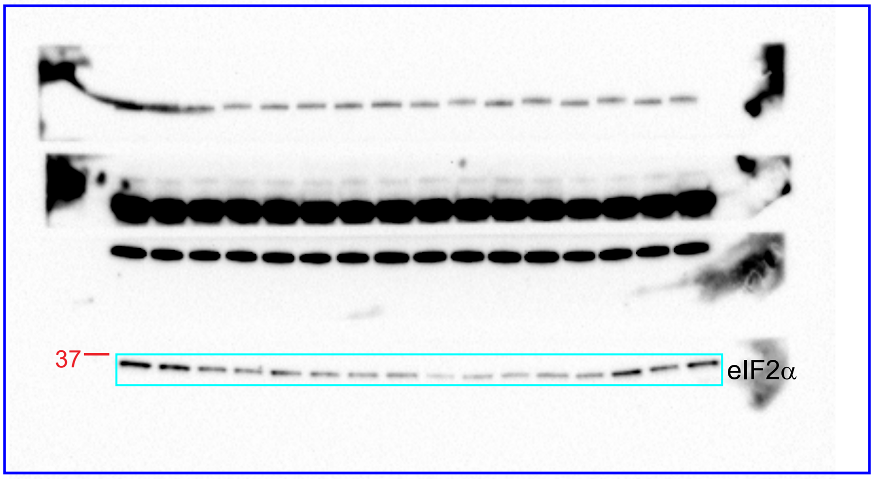

Figure panel γ–tubulin

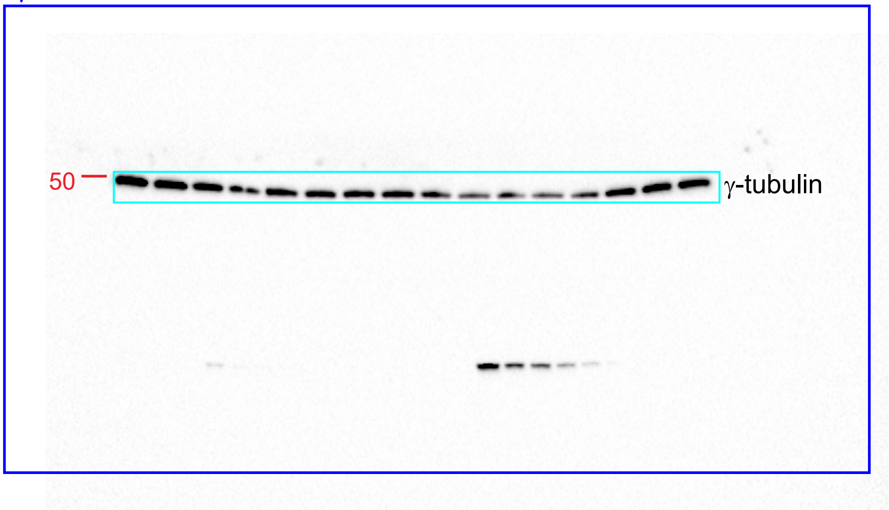

Figure S5 G

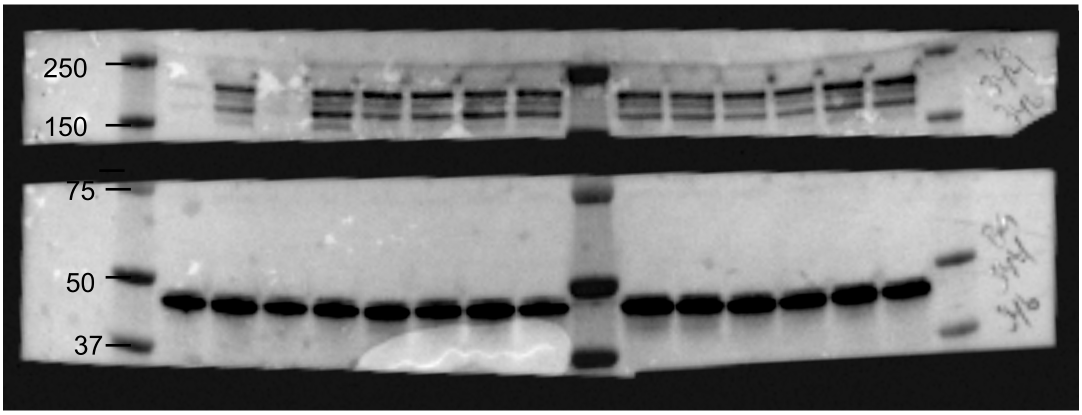

The blot was cut at the 150 and 75 kDa marker.
The upper part >150 kDa was probed with a GCN2 antibody.
The lower part <75 kDa was probed with a γ-tubulin antibody.

Figure panel GCN2

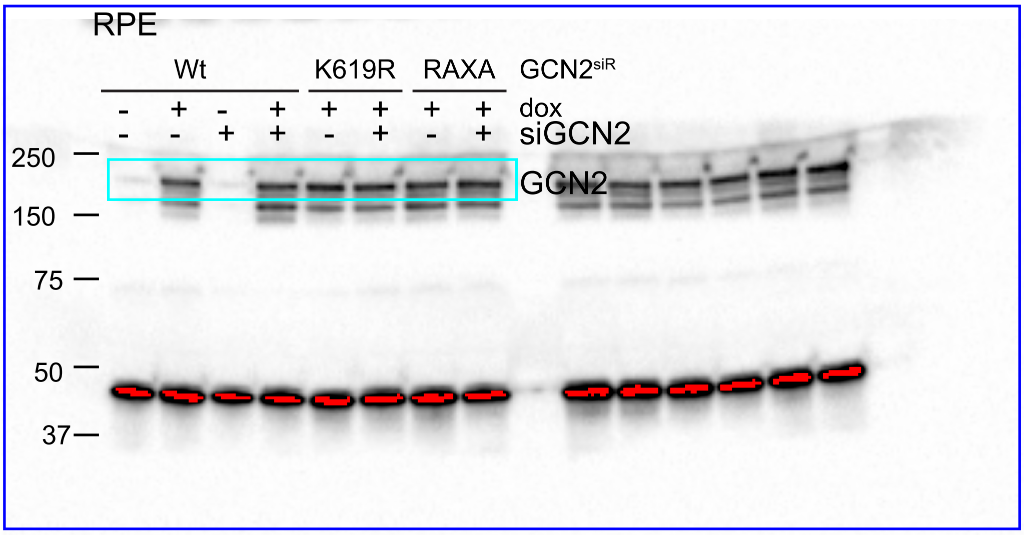

Figure panel γ-tubulin

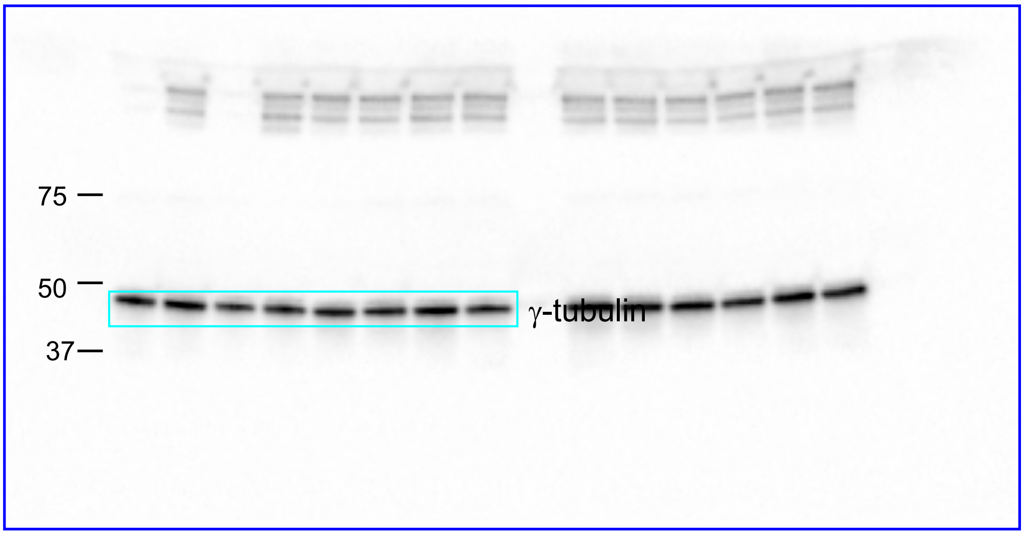

Figure S5 G, H hTert-RPE1 (G) or HeLa (H) cells transduced with doxycycline-inducible siRNA-resistant GCN2 carrying the indicated mutations were transfected with control or GCN2-targeting siRNA and incubated in the presence or absence of doxycycline as shown, to assess GCN2 levels by immunoblotting. γ-tubulin is shown as a loading control. Related to Fig 5 E, F.

Figure S5 H

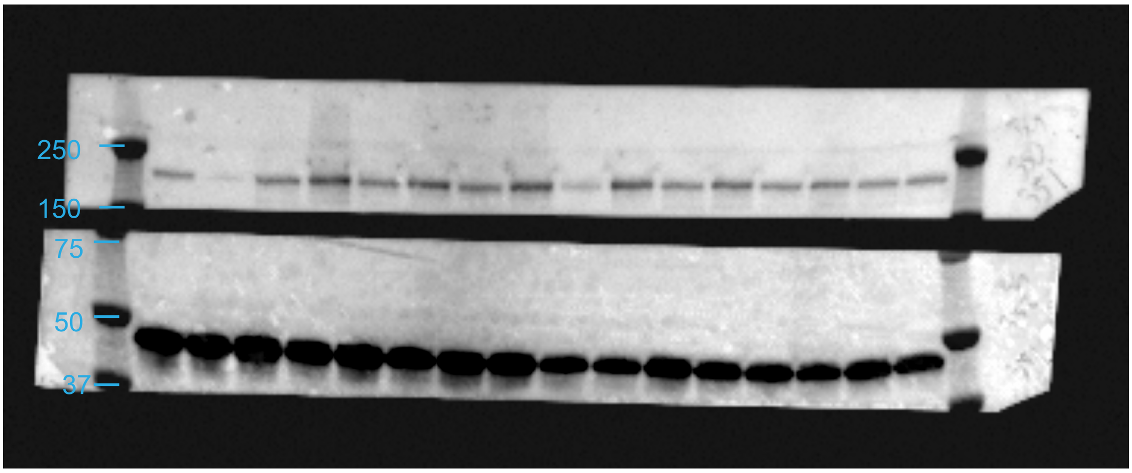

The blot was cut at the 150 and 75 kDa marker.
The upper part >150 kDa was probed with a GCN2 antibody.
The lower part <75 kDa was probed with a γ-tubulin antibody.

Figure panel GCN2

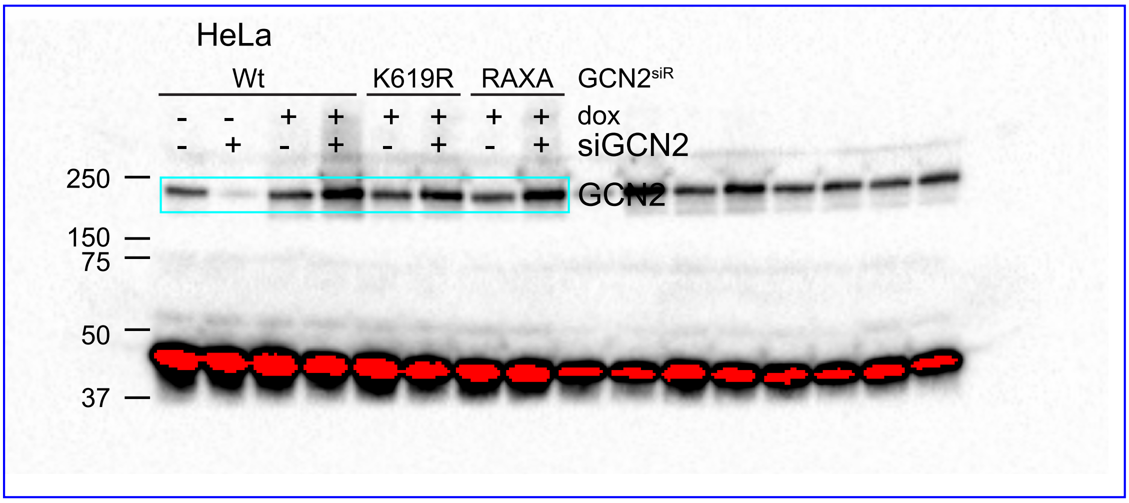

Figure panel γ-tubulin

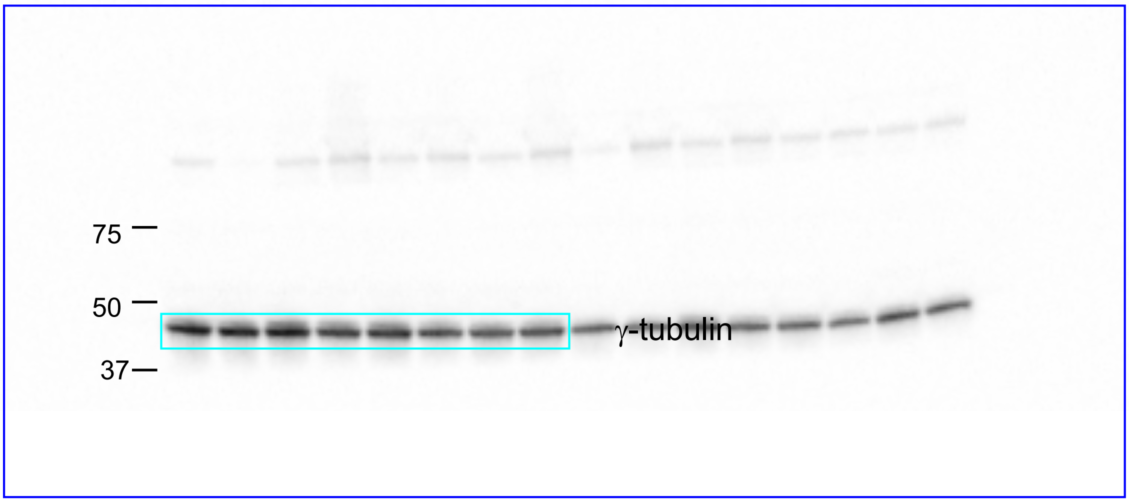

Figure S5 G, H hTert-RPE1 (G) or HeLa (H) cells transduced with doxycycline-inducible siRNA-resistant GCN2 carrying the indicated mutations were transfected with control or GCN2-targeting siRNA and incubated in the presence or absence of doxycycline as shown, to assess GCN2 levels by immunoblotting. γ-tubulin is shown as a loading control. Related to Fig 5 E, F.
