# Supplementary material for: Elevated GCN2 levels in cancer cells confer protection from mitotic stress and faster cell movement
Source: Cell Oncol (Dordr). 2026 May 11;49(4):92. doi: 10.1007/s13402-026-01214-5 (PMC13346413; doi:10.1007/s13402-026-01214-5)
Supplement: Supplementary file 10 — Supplementary Material 10 [file 13402_2026_1214_MOESM10_ESM.pdf]

Figure S1

A

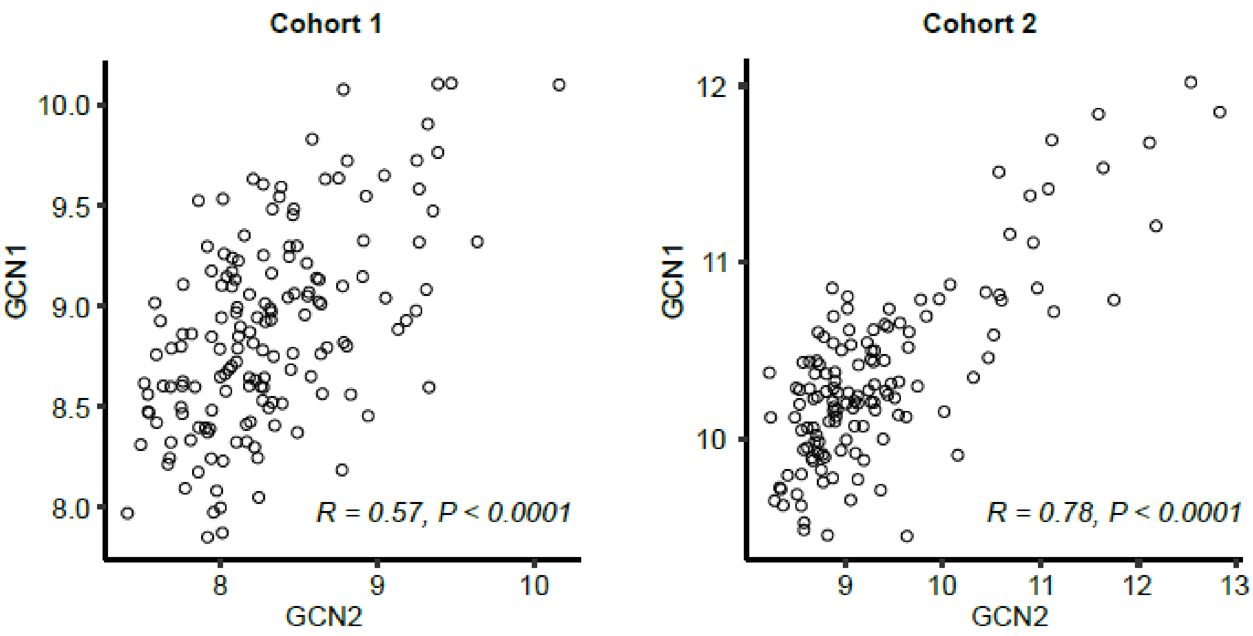
Cohort 1
10.0
9.5
9.0
8.5
8.0
GCN1
8
9
10
GCN2
R = 0.57, P < 0.0001
Cohort 2
12
11
10
GCN1
9
10
11
12
13
GCN2
R = 0.78, P < 0.0001

B

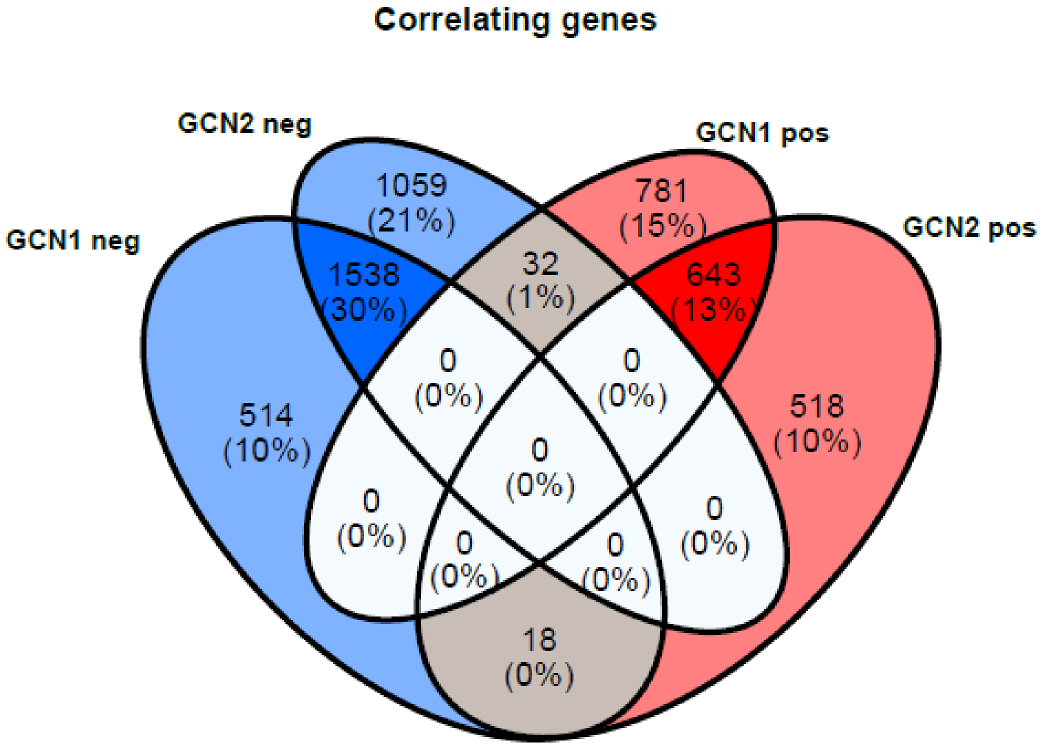
Correlating genes
GCN2 neg
GCN1 pos
GCN1 neg
GCN2 pos
1059
(21%)
781
(15%)
1538
(30%)
32
(1%)
643
(13%)
514
(10%)
518
(10%)
0
(0%)
18
(0%)

Figure S2

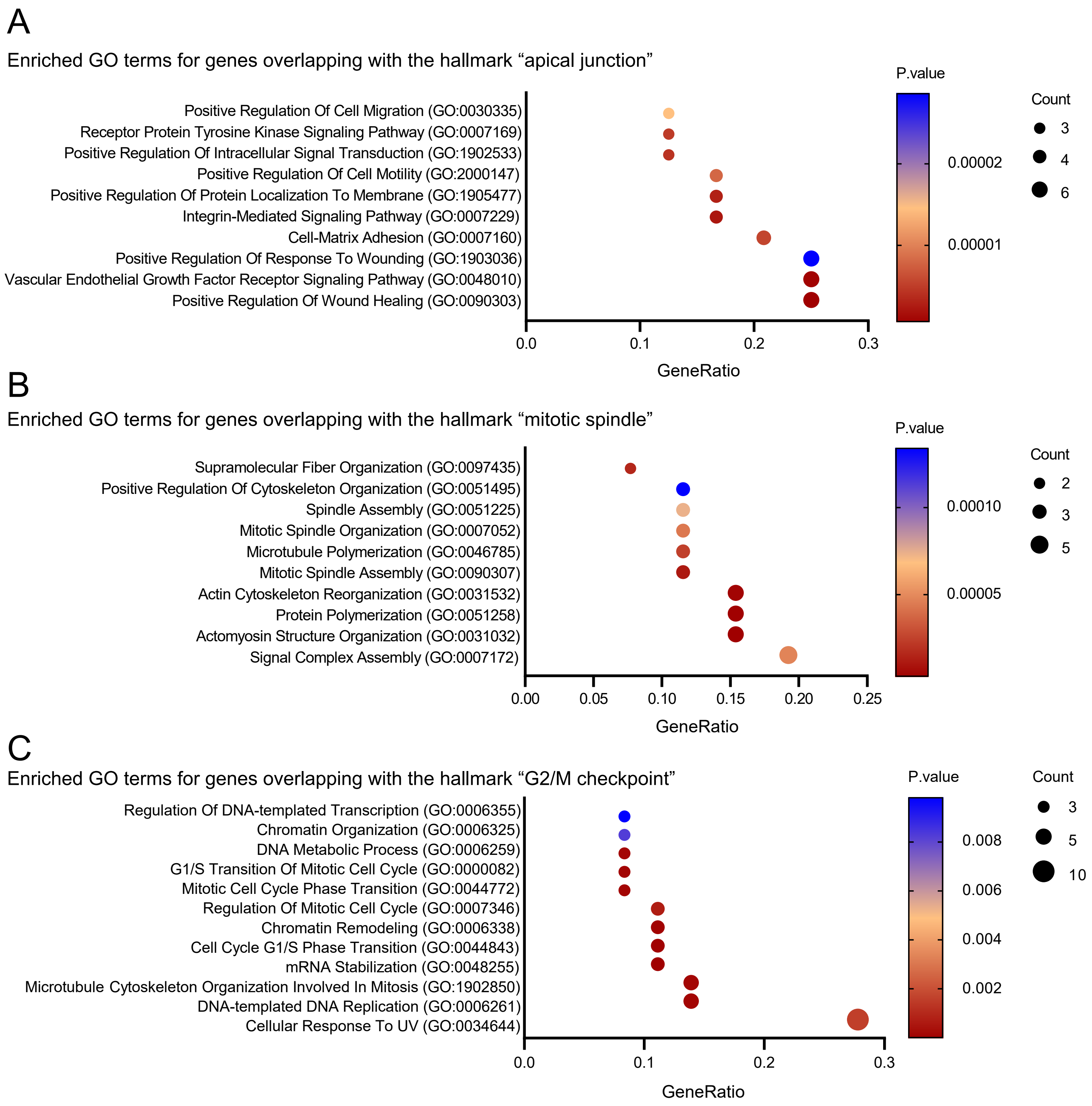
A
Enriched GO terms for genes overlapping with the hallmark "apical junction"
Positive Regulation Of Cell Migration (GO:0030335)
Receptor Protein Tyrosine Kinase Signaling Pathway (GO:0007169)
Positive Regulation Of Intracellular Signal Transduction (GO:1902533)
Positive Regulation Of Cell Motility (GO:2000147)
Positive Regulation Of Protein Localization To Membrane (GO:1905477)
Integrin-Mediated Signaling Pathway (GO:0007229)
Cell-Matrix Adhesion (GO:0007160)
Positive Regulation Of Response To Wounding (GO:1903036)
Vascular Endothelial Growth Factor Receptor Signaling Pathway (GO:0048010)
Positive Regulation Of Wound Healing (GO:0090303)
0.0
0.1
0.2
0.3
GeneRatio
P.value
0.00002
0.00001
Count
3
4
6
B
Enriched GO terms for genes overlapping with the hallmark "mitotic spindle"
Supramolecular Fiber Organization (GO:0097435)
Positive Regulation Of Cytoskeleton Organization (GO:0051495)
Spindle Assembly (GO:0051225)
Mitotic Spindle Organization (GO:0007052)
Microtubule Polymerization (GO:0046785)
Mitotic Spindle Assembly (GO:0090307)
Actin Cytoskeleton Reorganization (GO:0031532)
Protein Polymerization (GO:0051258)
Actomyosin Structure Organization (GO:0031032)
Signal Complex Assembly (GO:0007172)
0.00
0.05
0.10
0.15
0.20
0.25
GeneRatio
P.value
0.00010
0.00005
Count
2
3
5
C
Enriched GO terms for genes overlapping with the hallmark "G2/M checkpoint"
Regulation Of DNA-templated Transcription (GO:0006355)
Chromatin Organization (GO:0006325)
DNA Metabolic Process (GO:0006259)
G1/S Transition Of Mitotic Cell Cycle (GO:0000082)
Mitotic Cell Cycle Phase Transition (GO:0044772)
Regulation Of Mitotic Cell Cycle (GO:0007346)
Chromatin Remodeling (GO:0006338)
Cell Cycle G1/S Phase Transition (GO:0044843)
mRNA Stabilization (GO:0048255)
Microtubule Cytoskeleton Organization Involved In Mitosis (GO:1902850)
DNA-templated DNA Replication (GO:0006261)
Cellular Response To UV (GO:0034644)
0.0
0.1
0.2
0.3
GeneRatio
P.value
0.008
0.006
0.004
0.002
Count
3
5
10

Figure S3

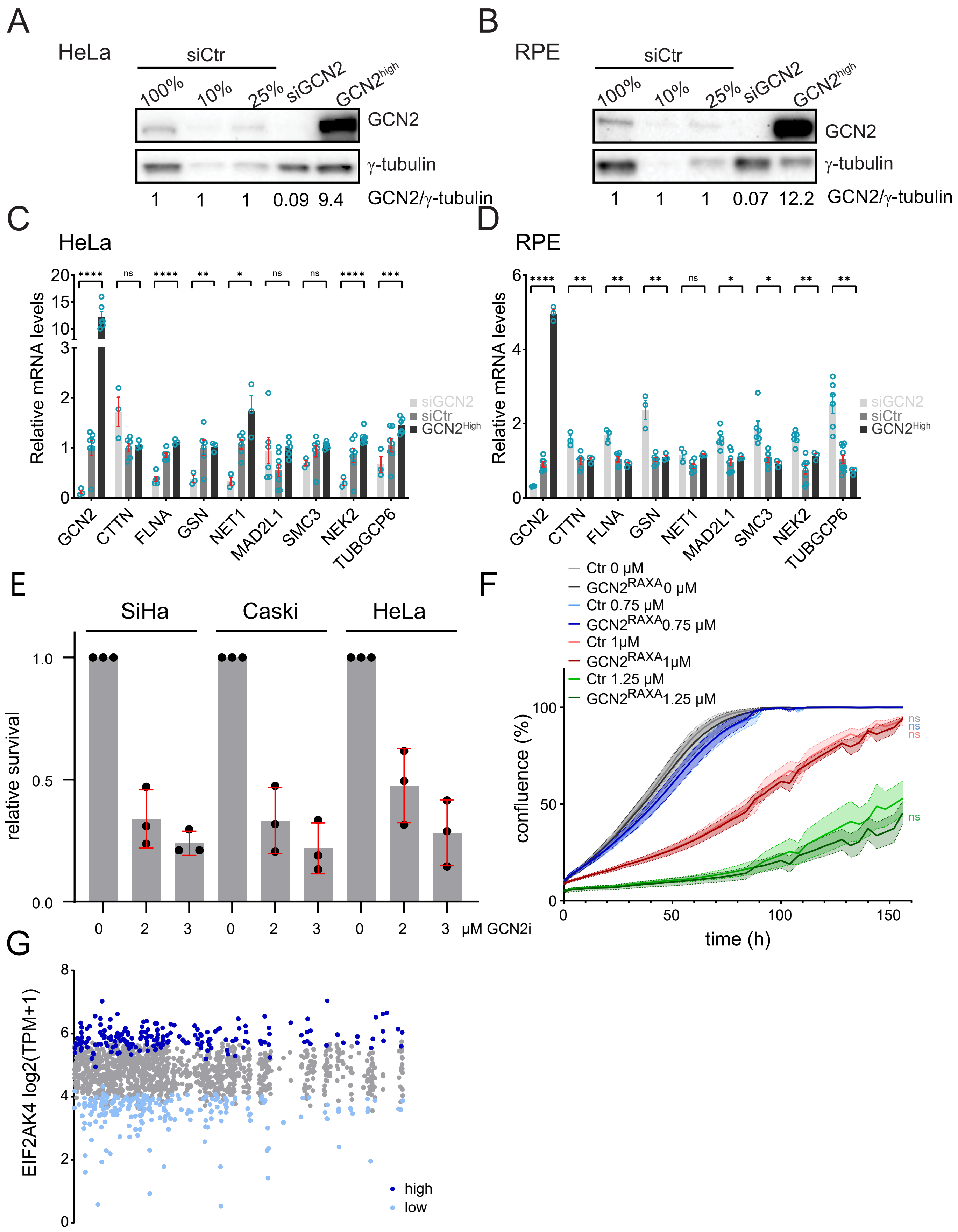

A
HeLa
siCtr
100%
10%
25%
siGCN2
GCN2high
GCN2
γ-tubulin
1 1 1 0.09 9.4
GCN2/γ-tubulin
B
RPE
siCtr
100%
10%
25%
siGCN2
GCN2high
GCN2
γ-tubulin
1 1 1 0.07 12.2
GCN2/γ-tubulin
C
HeLa
Relative mRNA levels
**** ns **** ** * ns ns **** ***
siGCN2
siCtr
GCN2High
GCN2
CTTN
FLNA
GSN
NET1
MAD2L1
SMC3
NEK2
TUBGCP6
D
RPE
Relative mRNA levels
**** ** ** ** ns * * ** **
siGCN2
siCtr
GCN2High
GCN2
CTTN
FLNA
GSN
NET1
MAD2L1
SMC3
NEK2
TUBGCP6
E
SiHa
Caski
HeLa
relative survival
0 2 3 0 2 3 0 2 3 μM GCN2i
F
Ctr 0 μM
GCN2RAXA 0 μM
Ctr 0.75 μM
GCN2RAXA 0.75 μM
Ctr 1μM
GCN2RAXA 1μM
Ctr 1.25 μM
GCN2RAXA 1.25 μM
confluence (%)
time (h)
ns
ns
ns
ns
G
EIF2AK4 log2(TPM+1)
high
low

Figure S4

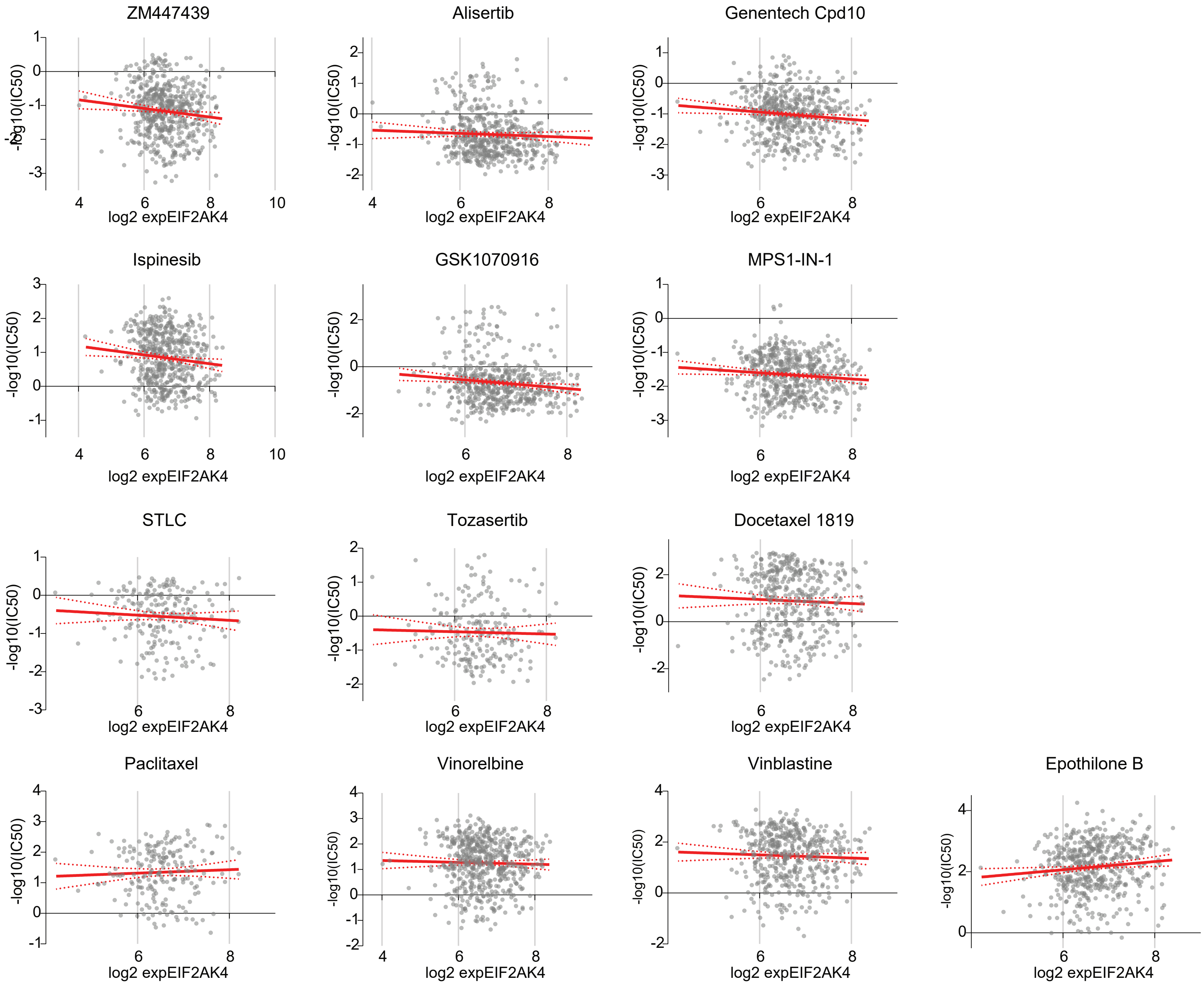
ZM447439
Alisertib
Genentech Cpd10
Ispinesib
GSK1070916
MPS1-IN-1
STLC
Tozasertib
Docetaxel 1819
Paclitaxel
Vinorelbine
Vinblastine
Epothilone B
-log10(IC50)
log2 expEIF2AK4

Figure S5

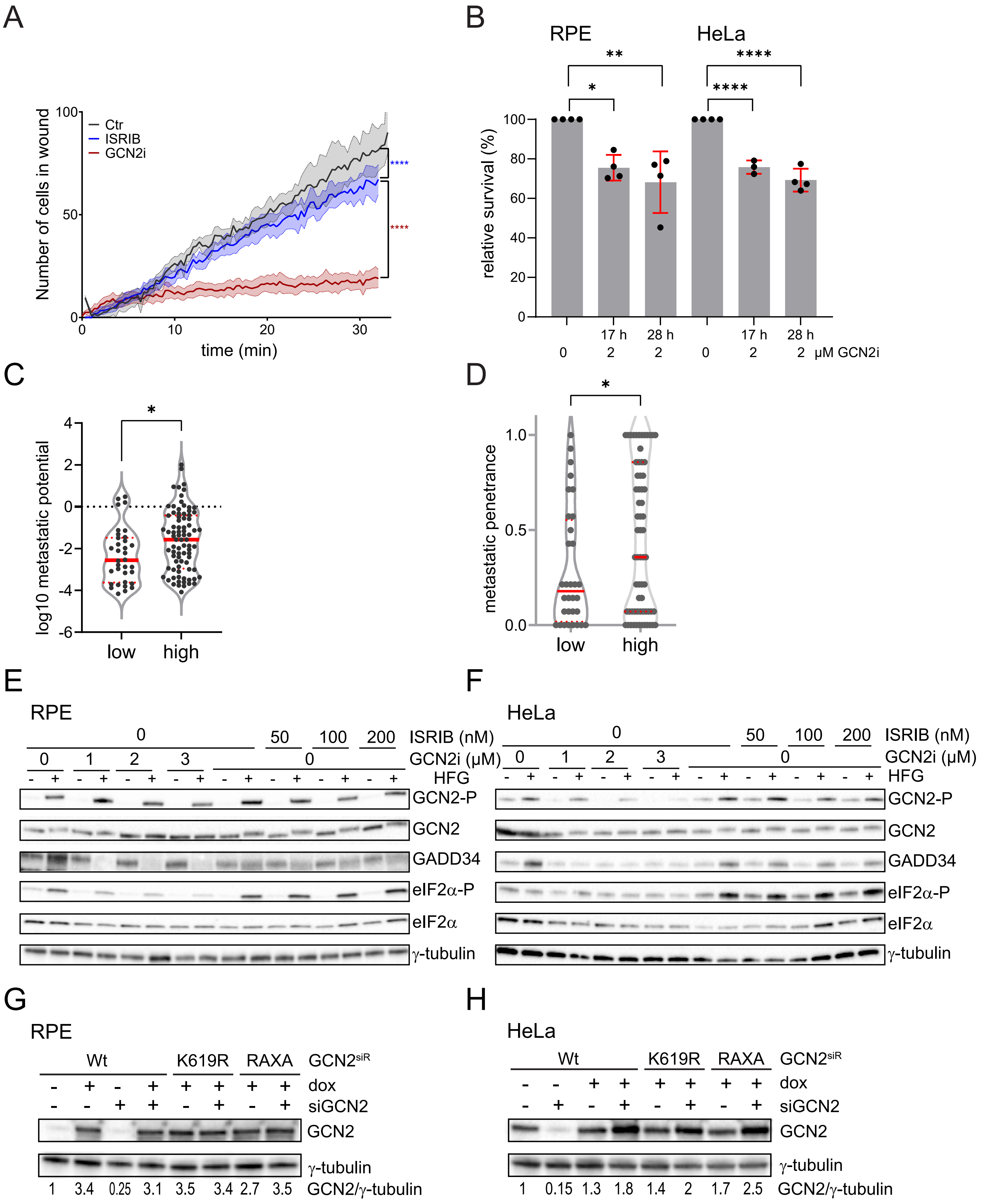
A
Number of cells in wound
100
50
0
Ctr
ISRIB
GCN2i
****
****
0
10
20
30
time (min)
B
RPE
HeLa
**
*
****
****
relative survival (%)
100
80
60
40
20
0
17 h
28 h
17 h
28 h
0
2
2
0
2
2
µM GCN2i
C
log10 metastatic potential
4
2
0
-2
-4
-6
*
low
high
D
metastatic penetrance
1.0
0.5
0.0
*
low
high
E
RPE
0
50
100
200
ISRIB (nM)
0
1
2
3
0
GCN2i (µM)
HFG
GCN2-P
GCN2
GADD34
eIF2α-P
eIF2α
γ-tubulin
F
HeLa
0
50
100
200
ISRIB (nM)
0
1
2
3
0
GCN2i (µM)
HFG
GCN2-P
GCN2
GADD34
eIF2α-P
eIF2α
γ-tubulin
G
RPE
Wt
K619R
RAXA
GCN2siR
dox
siGCN2
GCN2
γ-tubulin
1 3.4 0.25 3.1 3.5 3.4 2.7 3.5
GCN2/γ-tubulin
H
HeLa
Wt
K619R
RAXA
GCN2siR
dox
siGCN2
GCN2
γ-tubulin
1 0.15 1.3 1.8 1.4 2 1.7 2.5
GCN2/γ-tubulin

Figure S6

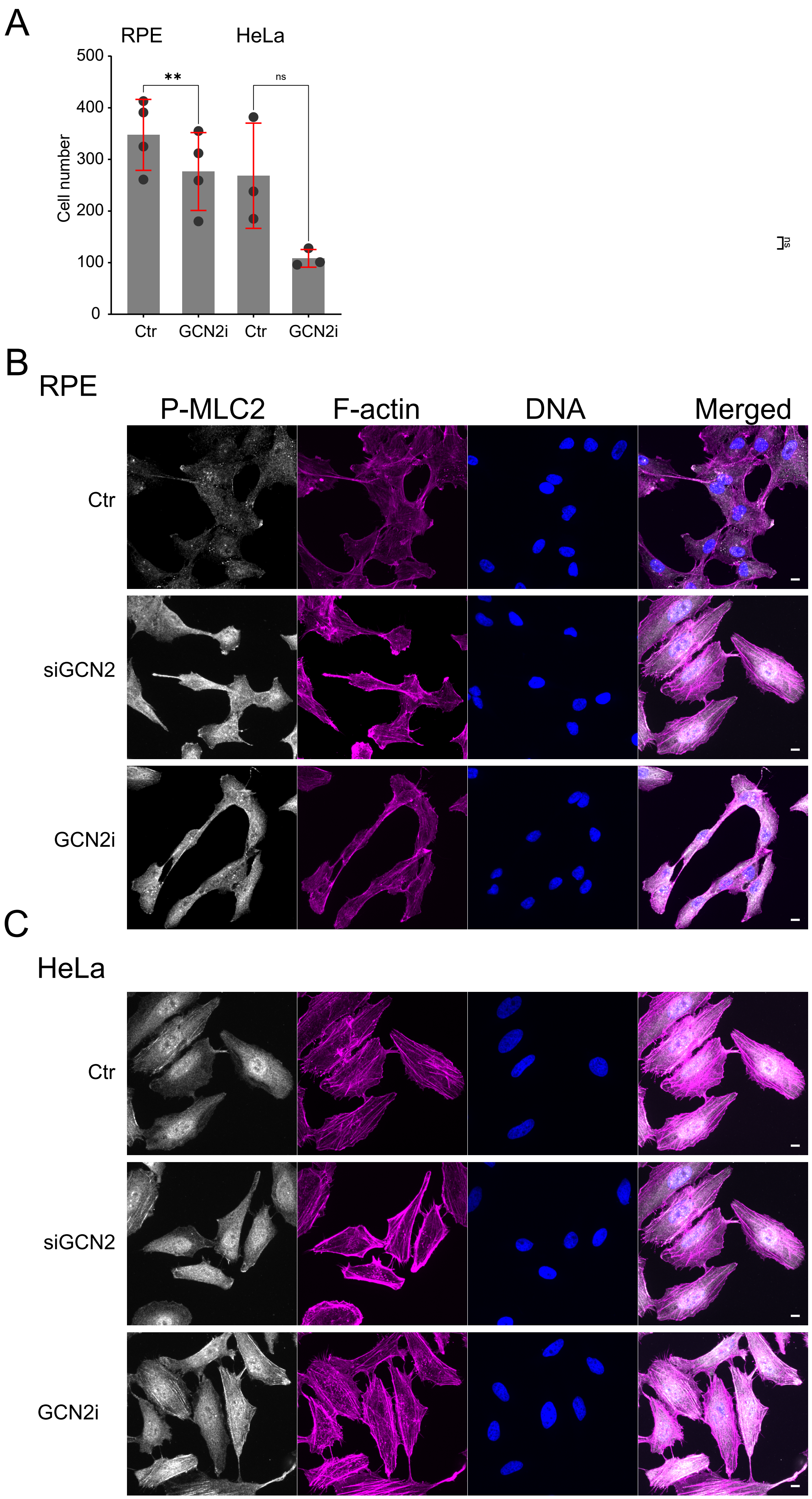
A
RPE
HeLa
500
400
300
200
100
0
Cell number
**
ns
Ctr
GCN2i
Ctr
GCN2i
ns
B
RPE
P-MLC2
F-actin
DNA
Merged
Ctr
siGCN2
GCN2i
C
HeLa
Ctr
siGCN2
GCN2i

Supplementary figure legends

Figure S1 A: Scatter plot of gene expression levels of GCN1 versus GCN2 in two cervical cancer patient cohorts. Left: cohort 1, n = 156. Right: cohort 2, n = 135. Correlation coefficient (R) and p-value from Spearman's rank correlation are indicated in plots

B: Venn diagram showing the overlap of GCN1- and GCN2- positively and negatively correlating genes. Number of genes and percent genes in each overlap are indicated. Gene symbols, correlation coefficients and p-values are shown in Table S1.

Figure S2 GO terms of the GCN2 correlating genes with an overlap with the hallmarks "apical junctions" (A), "mitotic spindle" (B), and "G2/M checkpoint" in Figure 2A. x axes show the ratio of genes of interest from the hallmark overlapping with the geneset in the GO term, compared to the total number of genes in the GO term. The analysis was performed at https://tnmplot.com/analysis/, the top 12 with the lowest p values are shown. Gene symbols are listed in Table S4. Related to Fig 1F

Figure S3 A, B Representative immunoblots to show GCN2 levels in the (**A**) HeLa and (**B**) hTert-RPE1 cell lines used in C and D. Cells were transfected with GCN2-targeting siRNA-s to deplete (siGCN2) or were engineered to stably overexpress GCN2 by lentiviral transduction ($GCN2^{high}$). To estimate the efficiency of depletion, different amounts (100%, 10% and 25%; corresponds to 30, 3 and 7.5 µg protein, respectively) of the lysate from cells transfected with control siRNA was loaded, along with 30 µg each of the transfected and the overexpressing samples. γ-tubulin is shown as a loading control.

C, D mRNA levels of selected transcripts in (**C**) HeLa and (**D**) hTert-RPE1 cells. mRNA levels were normalized to TBP and to GCN2 levels in the parental cell line. Each gene was analyzed in at least three independent experiments. The statistical analyses (unpaired t-test, Benjamini, Krieger and Yekutieli method) compared values between the depleted and overexpressing samples,

****p<0.0001, (D) p=0.0879 for CTTN, **p=0.0019 for GSN, *p=0.0111 for NET1, p=0.8585 for MAD2L1, ***p=0.0002 for SMC3, ***p=0.0009 for TUBGCP6, (E) **p=0.0096 for CTTN, **p=0.0025 for FLNA, **p=0.0088 for GSN, p=0.9396 for NET1, *p=0.0128 for MAD2L1, *p=0.0149 for SMC3,

**p=0.0037 for NEK2, **p=0.0020 for TUBGCP6.

E Inhibition of GCN2 leads to reduced cell survival in SiHa, Caski, and HeLa cells. The indicated cell lines were incubated in the presence of GCN2 inhibitor for 5 days. Cell survival was

assessed using the CCK8 assay. Data from three independent experiments are shown, mean and SD, one-way Anova, ****p<0.0001.

F HeLa cells transduced to express siRNA-resistant GCN2^{RAXA} were transfected with GCN2-targeting siRNA, grown in the presence of Eg5i at the indicated concentrations and observed in Incucyte. (E) Data shown are from four independent experiments. Non-linear regression. (E) p=0.5069 for 0 µM, p=0.9963 for 0.75 µM, p=0.4905 for1 µM, and p=0.3517 for 1.25 µM.

G Cell lines with the highest and lowest GCN2-expression levels (top and bottom 10% out of 1674 cell lines) in the DepMap database. The selected cell lines were used for the correlation analyses shown in Fig2F, G and Fig S5G, H.

Figure S4 The CellMiner database was used to download GCN2 gene expression data from the CTRP-Broad dataset and plot against -log10(IC50) values in the Sanger GDSC1 and 2 datasets. Higher −log10(IC50) indicates greater sensitivity. Spearman correlation coefficients and p values are: ZM447439 r= -0.121, p=0.0043; alisertib r=-0.148, p=0.0007; Genentech Cpd10 r=-0.106, p=0.0138; ispinesib r=-0.106 p=0.0139; GSK1070916 r= -0.121, p=0.0061; MPS1-IN-1 r=-0.106, p=0.0138; STLC r=-0.0598, p=0.3941; tozasertib r=-0.0227, p=0.3941; docetaxel r=-0.0645, p=0.1874; paclitaxel r=0.0529, p=0.2117; vinorelbine r=0.0231, p=0.5961; vinblastine r=-0.0682, p=0.1427 epothilone B r=0.121, p=0.0059

Figure S5 A Caski cells treated with 2 µM GCN2i or 200 nM ISRIB were seeded into Ibidi culture inserts and grown to confluence. After removal of the inserts the cells were observed by live-cell imaging. The number of cells migrating into the initial wound area is shown. Four independent experiments, mean and SEM are shown, non-linear regression, ****p<0.0001. Related to Fig 3 and 5A, B.

B, RPE and HeLa cells were seeded in the presence or absence of 2 µM GCN2i. Cell viability was assessed using the CCK8 assay after 17 or 28 hours of incubation. Data from four independent experiments, mean and SD are shown, one-way Anova, *p=0.0114, **p=0.0023 for RPE and ****p<0.0001 for HeLa.

C, D Metastatic potential (**C**) and penetrance (**D**) of the cell lines with low and high (10%, see Fig S3G) GCN2 expression. Mann Whitney test *p=0.0125 (G), Welch's t test *p=0.0355 (H)

E, F hTert-RPE1(C) and HeLa (D) cells were treated with 600 nM (**E**) or 60 nM (**F**) HFG in the presence of ISRIB or GCN2i as indicated, and GCN2 autophosphorylation, GADD34 induction and eIF2α phosphorylation were assessed by immunoblotting. GAPDH and γ-tubulin are shown as loading control. Related to Fig 5A, B.

G, H hTert-RPE1 (**G**) or HeLa (**H**) cells transduced with doxycycline-inducible siRNA-resistant GCN2 carrying the indicated mutations were transfected with control or GCN2-targeting siRNA and incubated in the presence or absence of doxycycline as shown, to assess GCN2 levels by immunoblotting. γ-tubulin is shown as a loading control. Related to Fig 5 E, F.

Figure S6 A Cells adhered 1 h (RPE) or 2h (HeLa) after seeding were stained with DAPI and cells were counted using NIS elements 6. Mean values and SD are shown, one-way Anova, ** p= 0.002.

B, C Cells of the indicated cell lines subjected to the indicated treatments were fixed 24 after seeding into Ibidi 8-well chambers with 4% formaldehyde, and stained with an antibody against PMLC (Cell Signaling #3674), phalloidin and DAPI. Representative images are shown. Scale bars represent 10 µM.
