# Supplementary material for: Elevated GCN2 levels in cancer cells confer protection from mitotic stress and faster cell movement
Source: Cell Oncol (Dordr). 2026 May 11;49(4):92. doi: 10.1007/s13402-026-01214-5 (PMC13346413; doi:10.1007/s13402-026-01214-5)
Supplement: Supplementary file 16 — Supplementary Material 16 [file 13402_2026_1214_MOESM16_ESM.docx]

Table S6 Number of genes identified in the proteomics datasets

Full gene lists and enrichment are shown in Table S5, genes enriched > 2x or >3X in the BioID and the PP1 interactome datasets are shown in Fig 6 F and G, respectively.

| Number of selected genes | 101 |  | 256 |  |
| --- | --- | --- | --- | --- |
| Overlap in BioID set | 70 |  | 127 |  |
| Enriched in BioID >2x | 13 |  | 19 |  |
| Found in PP1γ IP | 26 |  | 41 |  |
| siGCN2/siCtr >3 | 9 |  | 16 |  |
| siGCN2/siCtr <0.3 | 3 |  | 3 |  |
